# Chaining for accurate alignment of erroneous long reads to acyclic variation graphs

**DOI:** 10.1093/bioinformatics/btad460

**Published:** 2023-07-26

**Authors:** Jun Ma, Manuel Cáceres, Leena Salmela, Veli Mäkinen, Alexandru I Tomescu

**Affiliations:** Department of Computer Science, University of Helsinki, 00014 Helsinki, Finland; Department of Computer Science, University of Helsinki, 00014 Helsinki, Finland; Department of Computer Science, University of Helsinki, 00014 Helsinki, Finland; Department of Computer Science, University of Helsinki, 00014 Helsinki, Finland; Department of Computer Science, University of Helsinki, 00014 Helsinki, Finland

## Abstract

**Motivation:**

Aligning reads to a variation graph is a standard task in pangenomics, with downstream applications such as improving variant calling. While the vg toolkit [Garrison et al. (Variation graph toolkit improves read mapping by representing genetic variation in the reference. Nat Biotechnol 2018;36:875–9)] is a popular aligner of short reads, GraphAligner [Rautiainen and Marschall (GraphAligner: rapid and versatile sequence-to-graph alignment. Genome Biol 2020;21:253–28)] is the state-of-the-art aligner of erroneous long reads. GraphAligner works by finding candidate read occurrences based on *individually* extending the best seeds of the read in the variation graph. However, a more principled approach recognized in the community is to co-linearly chain *multiple* seeds.

**Results:**

We present a new algorithm to co-linearly chain a set of seeds in a string labeled acyclic graph, together with the first efficient implementation of such a co-linear chaining algorithm into a new aligner of erroneous long reads to acyclic variation graphs, GraphChainer. We run experiments aligning real and simulated PacBio CLR reads with average error rates 15% and 5%. Compared to GraphAligner, GraphChainer aligns 12–17% more reads, and 21–28% more total read length, on real PacBio CLR reads from human chromosomes 1, 22, and the whole human pangenome. On both simulated and real data, GraphChainer aligns between 95% and 99% of all reads, and of total read length. We also show that minigraph [Li et al. (The design and construction of reference pangenome graphs with minigraph. Genome Biol 2020;21:265–19.)] and minichain [Chandra and Jain (Sequence to graph alignment using gap-sensitive co-linear chaining. In: Proceedings of the 27th Annual International Conference on Research in Computational Molecular Biology (RECOMB 2023). Springer, 2023, 58–73.)] obtain an accuracy of <60% on this setting.

**Availability and implementation:**

GraphChainer is freely available at https://github.com/algbio/GraphChainer. The datasets and evaluation pipeline can be reached from the previous address.

## 1 Introduction

***Motivation.*** Variation graphs are a popular representation of all the genomic diversity of a population (Computational Pan-Genomics Consortium 2018, Eizenga *et al.* 2020, Miga and Wang 2021). While a single reference sequence can be represented by a single labeled path, a variation graph encodes each variant observed in the population via an “alternative” path between its start and end genomic positions in the reference labeled path. For example, the popular vg toolkit (Garrison *et al.* 2018) can build a variation graph from a reference genome and a set of variants, or from a set of reference genomes, while also supporting alignment of reads to the graph.

An interesting feature of a variation graph is that it allows for recombinations of two or more variants that are not necessarily present in any of the existing reference sequences used to build the graph. Namely, the paths of a variation graph now spell novel haplotypes recombining different variants, thus improving the accuracy of downstream applications, such as variant calling (Hurgobin and Edwards 2017, Garrison *et al.* 2018, Valenzuela *et al.* 2018, Hickey *et al.* 2020, Sirén *et al.* 2021). In fact, it has been observed (Garrison *et al.* 2018) that mapping reads with vg to a variation graph leads to more accurate results than aligning them to a single reference (since it decreases the reference bias). The alignment method of vg was developed for short reads (Garrison *et al.* 2018), and while it can run also on long reads, it decreases its performance (running time, memory usage, and alignment accuracy) significantly (Rautiainen and Marschall 2020). To the best of our knowledge, the only sequence-to-graph aligners tailored to long reads are SPAligner (Dvorkina *et al.* 2020), designed for assembly graphs, GraphAligner (Rautiainen and Marschall 2020), designed for both assembly and variation graphs, PaSGAL (Jain *et al.* 2019), designed for both short and long reads on acyclic graphs, the extension of AStarix for long reads (Ivanov *et al.* 2021) as well as minigraph (Li *et al.* 2020) and the recent minichain (Chandra and Jain 2023). GraphAligner is the state-of-the-art aligner for more-erroneous long reads (when mentioning erroneous long reads we refer to error rates typically observed in PacBio CLR reads. In our experiments we use 5% and 15% error rates to simulate reads) at the whole human pangenome level (highly variable whole human genome graphs of thousands of individuals), allowing for faster and more accurate alignments.

***Background.*** At the core of any sequence-to-graph aligner is the so-called *string matching to a labeled graph* problem, asking for a path of a node-labeled graph (e.g. a variation graph) whose spelling matches a given pattern. If allowing for approximate string matching under the minimum number of edits in the sequence, then the problem can be solved in quadratic time (Amir *et al.* 2000), and extensions to consider affine gap costs (Jain *et al.* 2020) and various practical optimizations were developed later. These practical optimizations were implemented into fast exact aligners such as PaSGAL (Jain *et al.* 2019), GraphAligner (Rautiainen *et al.* 2019, Rautiainen and Marschall 2020), and AStarix (Ivanov *et al.*2020, 2021).

Since, conditioned on SETH, no strongly subquadratic-time algorithm for edit distance on sequences can exist (Backurs and Indyk 2015), these sequence-to-graph alignment algorithms are worst-case optimal up to subpolynomial improvements. This lower bound holds even if requiring only *exact* matches, the graph is acyclic, i.e. a directed acyclic graph (DAG) (Equi *et al.* 2019, Gibney *et al.* 2021), and we allow any polynomial-time indexing of the graph (Equi *et al.* 2021). Recently, the bound was shown to hold for the special case of De Bruijn graphs (Gibney *et al.* 2022).

Given the hardness of this problem, current tools such as vg, GraphAligner, minigraph, and minichain employ various heuristics and practical optimizations to approximate the sequence-to-graph alignment problem, such as partial order alignment (Lee *et al.* 2002), as in the case of vg, seed-and-extend based on minimizers (Roberts *et al.* 2004), as in the case of GraphAligner, minigraph and minichain, and co-linear chaining in graphs (Mäkinen *et al.* 2019) as in the case of minigraph and minichain. In the case of GraphAligner, the aligner finds the minimizers of the read that have occurrences in the graph, clusters and ranks these occurrences, similarly to minimap (Li 2016), and then tries to extend the best clusters using an optimized *banded* implementation of the bit-parallel edit distance computation for sequence-to-graph alignment (Rautiainen *et al.* 2019). This strategy was shown to be effective in mapping erroneous long reads, since minimizers between erroneous positions can still have an exact occurrence in the graph. However, this strategy can still fail. For example, the seeds may be clustered in several regions of the graph and be separated in distance. Extending from one seed (cluster) through an erroneous zone to reach the next relatively accurate region would be hard in this case. Hence, for a long read, such an aligner may find several short alignments covering different parts of the read, but not a long alignment of the entire read. Furthermore, a seed may have many false alignments in the graph that are not useful in the alignment of the long read, but which can only be discarded when looking at its position compared to other seed hits.

A standard way to capture the global relation between the seed hits is through the *co-linear chaining* problem (Myers and Miller 1995), originally defined for two sequences as follows. Given a set of *anchors* consisting of pairs of intervals in the two sequences (e.g. various types of matching substrings, such as minimizers), the goal is to find a *chain* of anchors such that their intervals appear in the same order in both sequences. If the goal is to find a chain of maximum coverage in one of the sequences, the problem can be solved in time O(N log N) (Shibuya and Kurochkin 2003, Abouelhoda 2007, Mäkinen and Sahlin 2020), where *N* is the number of anchors. Co-linear chaining is successfully used by the popular read-to-reference sequence aligner minimap2 (Li 2018) and also in uLTRA (Mäkinen and Sahlin 2020), an aligner of RNA-seq reads. Moreover, recent results show connections between co-linear chaining and classical distance metrics (Mäkinen and Sahlin 2020, Jain *et al.* 2022).

The co-linear chaining problem can be naturally extended to a sequence and a labeled graph and has been previously studied for DAGs (Kuosmanen *et al.* 2018, Mäkinen *et al.* 2019), but now considering the anchors to be pairs of a path in the graph and a matching interval in the sequence. GraphAligner refers to chaining on DAGs as a principled way of chaining seeds (Rautiainen and Marschall 2020, p. 16), but does not implement co-linear chaining. Also SPAligner appears to implement a version of co-linear chaining between a sequence and an assembly graph. It uses the BWA-MEM library (Li 2013) to compute anchors between the long read and individual edge labels of the assembly graph, and then extracts “the heaviest chain of compatible anchors using dynamic programming” (Dvorkina *et al.* 2020, pp. 3–4). Their compatibility relation appears to be defined as in the co-linear chaining problem, and further requires that the distance between the anchor paths is relatively close to the distance between anchor intervals in the sequence. However, the overall running time of computing distances to check compatibility is quadratic (Dvorkina *et al.* 2020, Supplementary material). In the case of minigraph, the tool also computes seeds using minimizers and then applies a two-round co-linear chaining strategy to chain them. In the first round, it chains seeds within a node using a minimap2-like chaining algorithm, while in the second round it uses the chaining scores computed previously to chain the linear chains in the graph. Finally, the recent tool minichain showed how to improve minigraph’s chaining method by following a more principled approach on their chaining algorithm.

If the co-linear chaining problem is restricted to **character**-labeled DAGs, it can be solved in time O(kN log N+k|V|), after a preprocessing step taking O(k(|V|+|E|) log |V|) time (Mäkinen *et al.* 2019) when suffix–prefix overlaps between anchor paths are not allowed. Here, the input is a DAG G=(V,E) of *width k*, which is defined as the cardinality of a minimum-sized set of paths of the DAG covering every node at least once (a *minimum path cover*, or MPC). Thus, if *k* is constant, this algorithm matches the running time of co-linear chaining on two sequences, plus an extra term that grows linearly with the size of the graph. Even though there exists an initial implementation of these ideas tested on (small) RNA splicing graphs (Kuosmanen *et al.* 2018), it does not scale to large graphs, such as variation graphs, because of the k|V| term spent when aligning each read. Moreover, in the same publication, the authors show how to solve the general problem when suffix–prefix path overlaps of any length are allowed with an additional running time of $O(L{\;\text{log}\;}^{2}|V|)$ or O(L+#o) (*L* corresponds to the sum of the path lengths, whereas #o is the number of overlaps between paths), by using advanced data structures (FM-index, 2D range search trees, generalized suffix tree).

***Contributions.*** In this paper, we compute for the first time the width of variation graphs of all human chromosomes of thousands of individuals, which we observe to be at most 9 (see Supplementary Table S4) (although our graphs have high variability, it is worth noting that they are acyclic excluding structural variants such as repeat expansions, inversions, long deletions, and insertions), further motivating the idea of parameterizing the running time of the co-linear chaining problem on the width of the variation graph.

**Figure 1.**
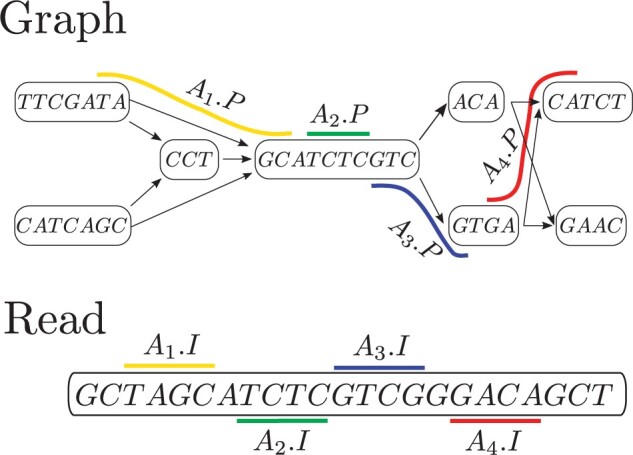
An illustrative example of Problem 2 consisting of a string labeled DAG, a read and a set of four anchors (paths in the graphs, paired with intervals in the read), shown here in different colors. The sequence $C=A_{1},A_{2},A_{3},A_{4}$ is a chain with cov(C)=16, and it is optimal since every other chain is subsequence of C. In particular, note that $A_{2}$ precedes $A_{3}$ because $A_{2}.y=11<A_{2}.x=12$ and $A_{2}.t$ reaches $A_{2}.t=A_{3}.s$, but $A_{2}.o_{t}=7<A_{3}.o_{s}=8$. If one-node suffix–prefix overlaps are not allowed, C is not a chain and an optimal chain (either $A_{1}$ or $A_{2}$ followed by $A_{4}$) would have coverage 8

We present the first algorithm solving co-linear chaining on string-labeled DAGs, when allowing one-node suffix–prefix overlaps between anchor paths. The reasons to consider such variation of the problem arise from practice. First, variation graphs used in applications usually compress unary paths, also known as unitigs (Kececioglu and Myers 1995), by storing the concatenation of their labels in a unique node representing them all. This not only allows to compress graph zones without variations, but also reduces the graph size, and therefore the running time of the algorithms that run on them. In Supplementary Tables S3 and S4, we show that typical variation graphs have 10 times more nodes in their character based representation (columns #Nodes and Labels bps). Second, as discussed before, allowing suffix–prefix overlaps of arbitrary length significantly increases the running time of the algorithm, and also requires the use of advanced data structures incurring in an additional penalty in practice. However, the important special nonoverlapping case in the character based representation translates into overlaps of at most one node in the corresponding string based representation (Fig. 1), which turns out to be easier to solve. Additionally, we show that allowing such overlaps suffices to solve co-linear chaining in graphs with cycles in the same running time as our solution for DAGs (Supplementary Data).

Our solution builds on the previous O(k(|V|+|E|) log |V|+kN log N) time solution (Mäkinen *et al.* 2019), to efficiently consider the one-node overlapping case, but without the need of advanced data structures (Section 2.3). Moreover, we show how to divide the running time of our algorithm into $O(k^{3}|V|+k|E|)$ for preprocessing the graph (Mäkinen *et al.* 2019, Cáceres *et al.* 2022), and O(kN log kN) for solving co-linear chaining (Supplementary Data). That is, for constant width graphs (such as variation graphs), our solution takes linear time to preprocess the graph plus O(N log N) time to solve co-linear chaining, removing the previous dependency on the graph size and matching the time complexity of the problem between two sequences. Since in practice *N* is usually much smaller than the DAG size, this result allows for a more efficient implementation of the algorithm in practice.

We then implement our algorithm for DAGs into GraphChainer, a new sequence-to-variation-graph aligner. On both simulated and real data, we show that GraphChainer allows for significantly more accurate alignments of erroneous long reads.

On simulated PacBio CLR reads (5% and 15% error rates), we classify a read as *correctly aligned* if the reported path overlaps (100⋅δ)% of the simulated region. We show that GraphChainer aligns ∼3% more reads than GraphAligner in all graphs tested and for every criterion 0<δ≤1. Moreover, if the criterion matches that of the average identity between the read and their ground truth, this difference increases to ∼6% on average. On real PacBio CLR reads, where the ground truth is not available, we classify a read as *correctly aligned* if the edit distance between the read and the reported sequence (without edits applied) is at most (100⋅σ)% of the read length. For criterion σ=0.3, GraphChainer aligns between 12% and 17% more real reads, of 21% and 28% more total length, on human chromosomes 1, 22, and the whole human pangenome. At the same criterion, GraphChainer aligns 95% of all reads or of total read length, while GraphAligner worsens its accuracy to <85% and 78%, respectively. We also run minigraph and minichain, and show that these tools obtain an accuracy of <60% for every graph, read set and criterion evaluated in our setting.

This increase in accuracy comes with a penalty in computational resource requirements: GraphChainer is ∼2–8× slower than GraphAligner and uses roughly two times the memory of GraphAligner in our real reads experiment. However, GraphChainer can run in parallel and its requirements even on the whole human pangenome graph are still within the capabilities of a modern high-performance computer. We also observe that the most time-intensive part of GraphChainer is obtaining the anchors for the input to co-linear chaining. This is currently implemented by aligning shorter fragments of the read using GraphAligner, however, given the modularity of this step, in the future other methods for finding anchors could be explored, such as using a fast short-read graph aligner, recent developments in MEMs (Rossi *et al.* 2022), or even recent advances of GraphAligner itself.

## 2 Materials and methods

### 2.1 Problem definition

Given a DAG G=(V,E), an *anchor A* is a tuple (P=(s,…,t),I=[x,y]), such that *P* is a path of *G* starting in *s* and ending in *t*, and I=[x,y] (with x≤y non-negative integers) is the interval of integers between *x* and *y* (both inclusive). We interpret *I* as an interval in the read matching the label of the path *P* in *G*. If *A* is an anchor, we denote by *A*.*P*, *A*.*s*, *A*.*t*, *A*.*I*, *A*.*x*, and *A*.*y* its path, path starting point, path endpoint, interval, interval starting point, and interval endpoint, respectively. Given a set of anchors $A=\{A_{1},\ldots ,A_{N}\}$, a *chain* C  *of anchors* (or just *chain* for short) is a sequence of anchors $A_{i_{1}},\ldots ,A_{i_{q}}$ of A, such that for all j∈{1,…,q−1}, $A_{i_{j}}$  *precedes* $A_{i_{j+1}}$, where the precedence relation corresponds to the conjunction of the precedence of anchor paths and of anchor intervals (hence *co-linear*). We will tailor the notion of precedence between paths and intervals for every version of the problem. We define the general co-linear chaining problem as follows:

**Problem 1** (Co-linear Chaining, CLC). *Given a DAG* G=(V,E)  *and a set* $A=\{A_{1},\ldots ,A_{N}\}$  *of anchors, find an* optimal *chain* $C=A_{i_{1}},\ldots ,A_{i_{q}}$  *according to some* optimization *criterion.*

Note that, although the final objective is to align a read to the variation graph, co-linear chaining can be defined (and solved) independent of the labels in these objects. However, in the case of string labeled graphs we will also need the exact offset $A.o_{s}$ in the label of *A*.*s*, where the string represented by the anchor path starts as well as the exact offset $A.o_{t}$ in the label of *A*.*t*, where the string represented by the anchor path finishes.

Co-linear chaining has been solved (Mäkinen *et al.* 2019) when the optimization criterion corresponds to maximizing the coverage of the read that is $\mathrm{cov}(C):=|\overset{q}{\underset{j=1}{\cup }}A_{i_{j}}.I|$, interval precedence is defined as integer inequality of interval endpoints ($A_{i_{j}}.y<A_{i_{j+1}}.y$), and path precedence is defined as strict reachability of path extremes (namely, $A_{i_{j}}.t$  *strictly* reaches $A_{i_{j+1}}.s$, i.e. with a path of at least one edge) in time O(k(|V|+|E|) log |V|+kN log N). If the precedence relation is relaxed to allow suffix–prefix overlaps of paths (two paths have a suffix–prefix overlap if the last *k* vertices of one (suffix) are exactly the last *k* vertices of the other (prefix), for some k>0), then their solution has an additional $O(L{\;\text{log}\;}^{2}|V|)$ or O(L+#o) (*L* corresponds to the sum of the path lengths, whereas #o is the number of overlaps between paths) term in the running time. We will show how to solve co-linear chaining on string labeled DAGs when path precedence allows a one-node suffix–prefix overlap. More precisely, we solve the following problem (Fig. 1):

**Problem 2** (One-node suffix–prefix overlap CLC). *Given a* string labeled *DAG* G=(V,E)  *and a set* $A=\{A_{1},\ldots ,A_{N}\}$  *of anchors, find a chain* $C=A_{i_{1}},\ldots ,A_{i_{q}}$  *maximizing* $\mathrm{cov}(C):=|\cup_{j=1}^{q}A_{i_{j}}.I|$*, such that for all* j∈{1,…,q−1}, $A_{i_{j}}$ precedes $A_{i_{j+1}}$*, meaning* $A_{i_{j}}.y<A_{i_{j+1}}.x$  *and* $A_{i_{j}}.t$  *reaches* $A_{i_{j+1}}.s$*, but if* $A_{i_{j}}.t=A_{i_{j+1}}.s$  *we also require that* $A_{i_{j}}.o_{t}<A_{i_{j+1}}.o_{s}$.

Note that changing the strict reachability condition from the path precedence by (normal) reachability allows one-node suffix–prefix overlaps between anchor paths (two paths have a one-node suffix–prefix overlap if the last vertex of one is exactly the first vertex of the other), and no more than that (since *G* is a DAG). Also note that our definition does not allow sequence overlaps (neither in the graph nor in the sequence), which will be reflected as a simplification in our algorithm.

### 2.2 Overview of the existing solution (without suffix–prefix overlaps)

The previous solution (Mäkinen *et al.* 2019) computes an MPC of *G* in time O(k(|V|+|E|) log |V|), which can be reduced by a recent result (Cáceres *et al.* 2022) to parameterized linear time $O(k^{3}|V|+|E|)$. Then, the anchors from A are sorted according to a topological order of its path endpoints (*A*.*t*) into $A_{1},\ldots ,A_{N}$. The algorithm uses a dynamic programming algorithm to compute for every j∈{1,…,N} the array:


$$C[j]=\max\{\mathrm{cov}(C)|C\;\text{is}\;a\;\text{chain of}\;\{A_{1},\ldots ,A_{j}\}\;\text{ending at}\;A_{j}\}.$$

Since chains (for this version of the problem) do not have suffix–prefix overlaps, they are all subsequences of $A_{1},\ldots ,A_{N}$, thus the optimal chain can be obtained by taking one of maximum C[*j*].

To compute C[*j*] efficiently, the algorithm maintains two rMq (range maximum query) data structures per path *i* in the MPC. One of the data structures, $D_{i}^{\cap }$, is used to compute the optimal chain whose last anchor interval overlaps the previous, and the other, Di∩, to compute the optimal chain whose last anchor interval does not overlap the previous. Since we do not consider sequence overlap, we will only use Di∩. The data structure of the *i*th path of the MPC, Di∩, stores the information on the *C* values (we refer to the original publication Mäkinen *et al.* (2019) for the exact information stored and queried in the data structure, or to our pseudocode in Algorithm 1) for all the anchors whose path endpoints belong to the *i*th path. Since the MPC, by definition, covers all the vertices of *G*, to compute a particular *C*[*j*] it suffices to query all the data structures that contain anchors whose path endpoint reaches $A_{j}.s$ (Mäkinen *et al.* 2019, Observation 1), which is done through the *forward propagation links* (we refer to the original publication Mäkinen *et al.* [2019] for the exact definition and construction algorithm for the *forward propagation links*, or to our pseudocode in Algorithm 1).

More precisely, the vertices *v* of *G* are processed in topological order:

**Step 1, update structures:** for each anchor $A_{j}$ whose path endpoint is *v*, and for each *i* such that *v* belongs to the *i*th path of the MPC, the data structure of the *i*th path is updated with the information of *C*[*j*].

**Step 2, update *C* values:** for each forward link (u,i) from *v* (i.e. *u* is the last vertex, different from *v*, that reaches *v* in the *i*th path), and each anchor $A_{j}$ whose path starting point is *u*, the entry *C*[*j*] is updated with the information stored in the data structure of the *i*th path.

Finally, since each data structure is queried and updated O(kN) times, and since these operations can be implemented in O(log N) time, the running time of the two steps for the entire computation is O(kN log N) plus O(k|V|) for scanning the forward links.

### 2.3 One-node suffix–prefix overlaps

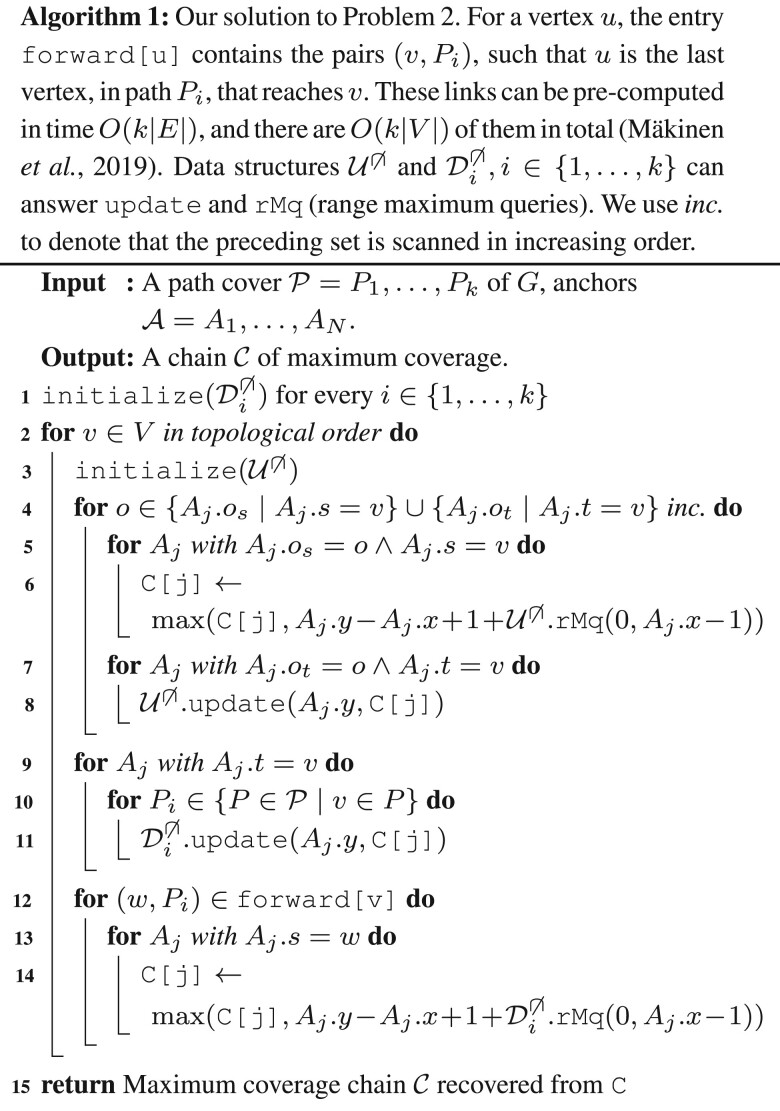

When trying to apply the approach from Section 2.2 to solve Problem 2 we have to overcome some problems. First, it no longer holds that every possible chain is a subsequence of $A_{1},\ldots ,A_{N}$ (the input A sorted by topological order of anchor path endpoints): an anchor *A* whose path is a single vertex *v* can be preceded by another anchor $A^{\prime }$ whose path ends at *v*, however, *A* could appear before $A^{\prime }$ in the order (e.g. $A_{1}$ and $A_{2}$ in Fig. 1 could have appear in another order). To solve this issue we solve ties of path endpoints (*A*.*t*) by further comparing offsets in the string label of the path endpoint ($A.o_{t}$). As such, anchors appearing after cannot precede anchors appearing before in the order, and the optimal chain can be obtained by computing the maximum *C*[*j*]. The sorting of anchors by this criterion can be done in O(N log N) time as it just corresponds to a sorting of the *N* anchors with a different comparison function, thus maintaining the asymptotic running time of O(k(|V|+|E|) log |V|+kN log N).

A second problem is that Step 1 assumes that *C*[*j*] contains its final value (and not an intermediate computation). Since suffix–prefix path overlaps were not allowed, this was a valid assumption in the previous case, however, because one-node suffix–prefix overlaps are now allowed, the chains whose $A_{j}.P$ has a suffix–prefix overlap of one node (with the previous anchor) are not considered. We fix this issue by adding a new Step 0 before Step 1 in each iteration. Step 0 includes (into the computation of *C*[*j*]) those chains whose last anchors have a one-node suffix–prefix overlap, before applying Steps 1 and 2. Step 0 uses one data structure (one in total, and is reinitialized in every Step 0), U∩, that maintains the information of the *C* values for all anchors whose paths ends at *v* (the currently processed node). In this case, the information in the data structure is not propagated through forward links (done in Step 2 as before), but instead this information is used to update the *C* values of anchors whose paths start at *v*.

More precisely, for every anchor $A_{j}$ whose path starts or ends at *v*, in increasing order of $A_{j}.o_{s}$ and $A_{j}.o_{t}$, respectively, we do:

**Step 0.1, update *C* value:** if the starting point of $A_{j}$’s path is *v*, then we update *C*[*j*] with the information stored in the data structures.

**Step 0.2, update the structure:** if the endpoint of $A_{j}$’s path is *v*, then we update the data structure with the information of *C*[*j*].

Note that in this case the update of the *C* value comes before the update of the data structures to ensure that single node anchor paths are chained correctly. Moreover, to avoid chaining two anchors *A* and $A^{\prime }$ with $A.t=A^{\prime }.s$ and $A.o_{t}=A^{\prime }.o_{s}$, we process all anchors with the same offset value together, i.e. we first apply Step 0.1 to all such anchors and then Step 0.2 to all such anchors. Algorithm 1 shows the pseudocode of the algorithm. Since every anchor requires at most two operations in the data structure the running time of Step 0 during the whole algorithm is O(N log N), thus maintaining the asymptotic running time of O(k(|V|+|E|) log |V|+kN log N).

In the Supplementary Data, we explain how to adapt Algorithm 1 to obtain $O(k^{3}|V|+k|E|)$ time for preprocessing and O(kN log kN) time for solving co-linear chaining. Also in the Supplementary Data, we show a slight variation of Problem 2 and Algorithm 1 to solve co-linear chaining on cyclic graphs.

### 2.4 Implementation

We implemented our algorithm to solve Problem 2 inside a new aligner of long reads to acyclic variation graphs, GraphChainer. Our C++ code is built on GraphAligner’s codebase. Moreover, GraphAligner’s alignment routine is also used as a blackbox inside our aligner as explained next (see Supplementary Fig. S4 for an overview).

To obtain anchors, GraphChainer extracts *fragments* of length ℓ=colinear-split-len (default 35, experimentally set) from the long read. By default GraphChainer splits the input read into nonoverlapping substrings of length ℓ each, which together cover the read. Additionally, GraphChainer can receive an extra parameter s=sampling-step (or just step for short), which samples a fragment every ⌈s×ℓ⌉ positions instead. That is, if s>1, the fragments do not fully cover the input read, and if s≤1−1/ℓ, fragments are overlapping. Having fewer fragments to align (bigger step) decreases the running time but also the accuracy. These fragments are then aligned with GraphAligner to the variation graph (all calls to GraphAligner are implemented internally, in the same binary of GraphChainer), with default parameters; for each alignment reported by GraphAligner, we obtain an anchor made up of the reported path in the graph (including the offsets in the first and last nodes $A.o_{s},A.o_{t}$), and the fragment interval in the long read. (We ignore the alignment score returned by GraphAligner. Other aligners such as minigraph and minichain consider this score as part of the optimization criterion of co-linear chaining.)

Having the input set of anchors, GraphChainer then solves Problem 2. The MPC index is computed with the O(k(|V|+|E|) log |V|) time algorithm (Mäkinen *et al.* 2019) for its simplicity, where for its max-flow routine we implemented Dinic’s algorithm (Dinic 1970). The rMq (range maximum query) data structures that our algorithm maintains per MPC path are supported by our own implementation of a treap (Seidel and Aragon 1996).

After the co-linear chaining algorithm outputs a maximum-coverage chain $A_{i_{1}},\ldots ,A_{i_{q}}$, GraphChainer connects the anchor paths to obtain a longer path, which is then reported as the answer. More precisely, for every j∈{1,…,q−1}, GraphChainer connects $A_{i_{j}}.t$ to $A_{i_{j+1}}.s$ by a shortest path (in the number of nodes). Such a connecting path exists by the definition of precedence in a chain, and it can be found by running a breadth-first search (BFS) from $A_{i}.t$. A more principled approach would be to connect consecutive anchors by a path minimizing edit distance, or to consider such distances as part of the optimization function of co-linear chaining. We use BFS for performance reasons.

Next, since our definition of co-linear chaining maximizes the coverage of the input read, it could happen that, in an erroneous chaining of anchors, the path reported by GraphChainer is much longer than the input read. To control this error, GraphChainer splits its solution whenever a path joining consecutive anchors is longer (label-wise) than some parameter g=colinear-gap (default 10 000, in the order of magnitude of the read length), and reports the longest path after these splits.

Finally, GraphChainer uses edlib (Šošić and Šikić 2017) to compute an alignment between the read and the path found, and to decide if this alignment is better than the (best) alignment reported by GraphAligner. Therefore, GraphChainer can be also viewed as a refinement of GraphAligner’s alignment results.

## 3 Experiments

We run several erroneous long read to variation graph alignment experiments, and compare GraphChainer (v1.0.2) against GraphAligner (v1.0.13), the state-of-the-art aligner of long reads to (pangenome level) variation graphs. We also test the performance of minigraph (v0.20) and minichain (v1.0), which also exploit co-linear chaining. We excluded SPAligner since this is tailored for alignments to assembly graphs. We also excluded PaSGAL and the recent extension of AStarix for long reads, since these aligners are tailored to find optimal alignments and they were three orders of magnitude slower than GraphChainer in our smallest dataset.

### 3.1 Datasets

***Variation graphs.*** We use two (relatively) small variation graphs, two chromosome-level variation graphs, one whole human genome variation graph and two other whole human genome variation graphs used in the experiments of minichain. The small graphs, LRC and MHC1 (Jain *et al.* 2019), correspond to two of the most diverse variation regions in the human genome (Dilthey *et al.* 2015, Sibbesen *et al.* 2018). The chromosome-level graphs, Chr22 and Chr1 (human chromosomes 22 and 1, respectively), were built with the vg toolkit using GRCh37 as the reference, and variants from the 1000 Genomes Project phase 3 release (Clarke *et al.* 2017). We use Chr22 to replicate GraphAligner’s results (Rautiainen and Marschall 2020), and consider Chr1 since it is one of the most complex variation graphs of the human chromosomes (see Supplementary Table S4). The whole human genome graph, AllChr, corresponds to the union graph of all chromosome variation graphs, each built as described before. Finally, we use graphs 10H and 95H created for the experiments of minichain (Chandra and Jain 2023) using minigraph and 10 and 95 publicly available haplotype-resolved human genome assemblies.

Note that acyclic variation graphs (built as above) span all genomic positions, i.e. do not collapse repeats like, e.g. de Bruijn graphs. As such, the aligner does not perform extra steps to identify the corresponding repeat of an alignment but instead they are solved by the alignment task itself.

***Simulated reads.*** For each of the previous graphs, we sample a reference sequence and use it to simulate a PacBio CLR read dataset of 15× coverage and average error rate of 15% and 5% (only 5% error rate for the case of 10H and 95H to replicate minichain’s experimental setting) with the package Badread (Wick 2019). We use 1× coverage in the case of AllChr. To build the reference sequence we first sample a source-to-sink path from the graph by starting at a source, and repeatedly choosing an out-neighbor of the current node uniformly at random, until reaching a sink; finally, we concatenate the node labels on the path. The ambiguous characters on the simulated reference sequence are randomly replaced by one of its indicated characters. For each simulated read, we know its original location on the sampled reference sequence, which can be mapped to its ground truth location on the graph.

***Real reads on chromosome-level graphs and whole human genome graph.*** For the chromosome-level graphs, we also used the whole human genome PacBio CLR Sequel data from HG00733 (SRA accession SRX4480530). (This is the same read set used by GraphAligner’s experiments [Rautiainen and Marschall 2020, p. 7] but without the subsampling to 15× coverage.) We first aligned all the reads against GRCh37 with minimap2 and selected only the reads that are aligned to chromosome 22 and 1, respectively, with at least 70% of their length, and have no longer alignments to other chromosomes. This filtering leads to 56× and 79× coverage on Chr22 and Chr1, respectively. For AllChr, we performed a uniformly random sample of all reads in the dataset to obtain 1× coverage. Supplementary Table S4 shows more statistics of the variation graphs and read sets.

### 3.2 Evaluation metrics and experimental setup

For each read, the aligners output an alignment consisting of a path in the graph and a sequence of edits to perform on the node sequences. We call this path the *reported path* and the concatenation of the node sequences without the edits the *reported sequence*. (Both objects, as well as the ground truth path/sequence, exclude the offsets of the first and last nodes.) Aligners return *mapping quality scores*, however, to normalize the comparison of results, we will use our own metrics to classify if an alignment was successful.

On simulated data, we classify a read as *correctly aligned* if the overlap (in genomic sequences) between the reported path and the ground truth path is at least (100⋅δ)% of the length of the ground truth sequence, where 0<δ≤1 is a given threshold. As another criterion, we consider a read correctly aligned if the edit distance between the reported sequence and ground truth sequence is at most (100⋅σ)% of the length of the ground truth sequence, where 0<σ≤1 is another given threshold. On real data (also on simulated data to show the relation between the criteria. This can be found in the Supplementary Data), where the ground truth is not available, we consider a read correctly aligned if the edit distance between the reported sequence and read is at most (100⋅σ)% of the read length.

Since the reads have varying sizes, we also computed the *good length*, defined as the total length of correctly aligned reads divided by the total read length, for every criterion and threshold considered.

All experiments were conducted on a server with AMD Ryzen Threadripper PRO 3975WX CPU with 32 cores and 504GB of RAM. All aligners were run using 10 threads for all datasets. Time and peak memory usage of each program were measured with the GNU time command. The commands used to run each tool are listed in the Supplementary Data. Our code, datasets and pipeline can be found at https://github.com/algbio/GraphChainer.

### 3.3 Results and discussion

#### 3.3.1 Comparison with GraphAligner

**Figure 2.**
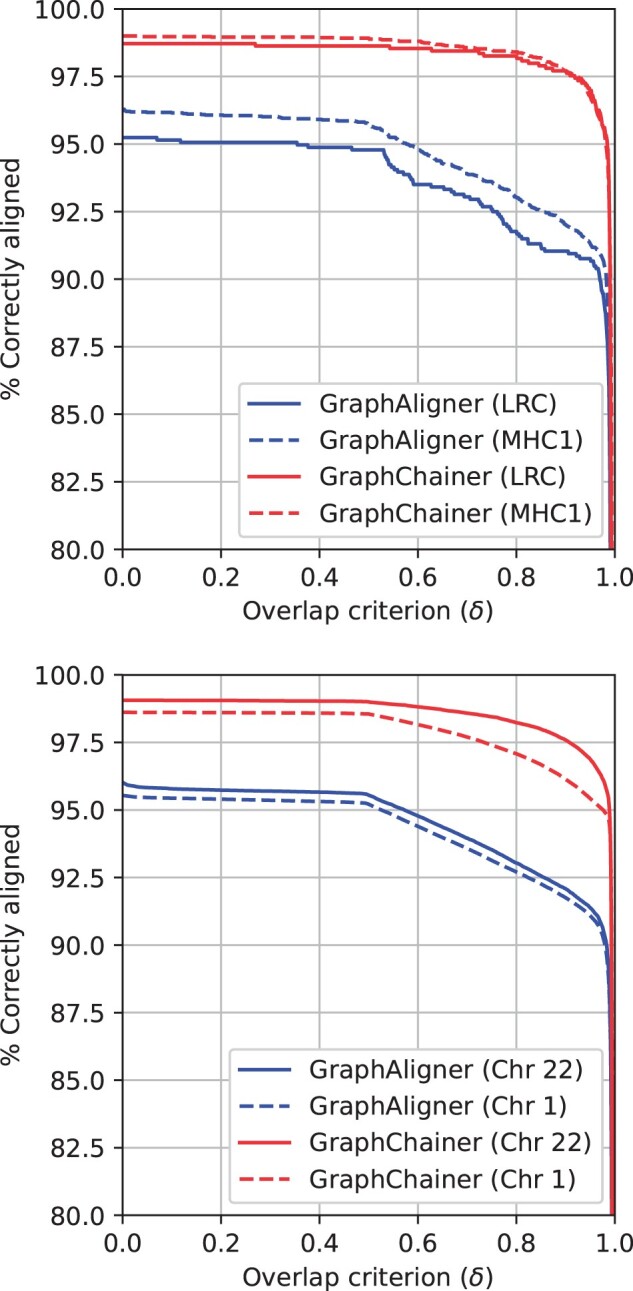
Correctly aligned reads w.r.t. overlap with ground truth on the simulated read sets for LRC (top solid), MHC1 (top dashed), Chr22 (bottom solid), Chr1 (bottom dashed) and AllChr (bottom dotted). Plots for minigraph and minichain can be found in the Supplementary Data

***Simulated reads with 15% error rate.*** For criterion δ=0.85, that is matching the average identity of the simulated reads, GraphChainer has at least 4–5% more correctly aligned reads, and at the weaker criterion δ=0.1, used in GraphAligner’s evaluation (Rautiainen and Marschall 2020, p. 3), GraphChainer correctly aligns 3–4% more reads (Table 1), which is true for every criterion δ>0 (Fig. 2). Moreover, we observe that the accuracy of GraphAligner drops below 95% for δ>0.6, whereas this does not happen to GraphChainer until δ>0.98. Similar results are obtained when measuring good length and using edit distance criteria (Supplementary Tables S9, S11, and S13 and Supplementary Figs S5, S6, S9, S10, S13, S14, S17, and S18).

**Table 1.** Correctly aligned reads with respect to the overlap for δ∈{0.1,0.85} (i.e. the overlap between the reported path and the ground truth is at least 10% or 85% of the length of the ground truth sequence, respectively) for the simulated read sets with 15% error rate.^a^

Graph	Aligner	Correctly aligned	
		δ=0.1	δ=0.85
LRC	GraphChainer	98.72% (+3.75%)	97.90% (+7.32%)
	GraphAligner	95.15%	91.22%
	minigraph	29.09%	7.04%
	minichain	6.40%	0.82%
MHC1	GraphChainer	98.98% (+2.94%)	98.17% (+6.11%)
	GraphAligner	96.15%	92.52%
	minigraph	30.84%	10.21%
	minichain	2.44%	0.35%
Chr22	GraphChainer	99.06% (+3.42%)	98.00% (+5.88%)
	GraphAligner	95.78%	92.56%
	minigraph	48.84%	30.99%
	minichain	31.17%	26.80%
Chr1	GraphChainer	98.61% (+3.32%)	96.68% (+4.79%)
	GraphAligner	95.44%	92.26%
	minigraph	36.82%	15.74%
	minichain	10.84%	8.26%
AllChr	GraphChainer	98.64% (+3.50%)	96.51% (+5.12%)
	GraphAligner	95.30%	91.81%
	minigraph	26.81%	7.21%
	minichain	2.28%	1.38%

a Percentages in parentheses are relative improvements w.r.t. GraphAligner.

**Figure 3.**
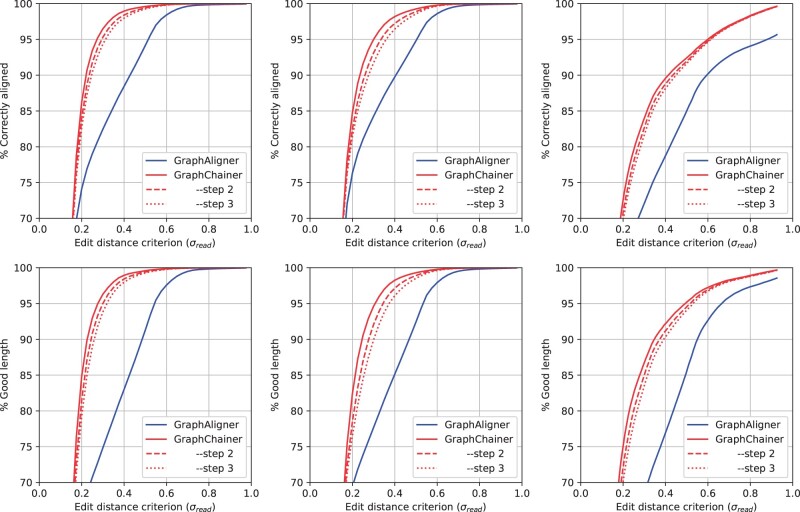
Correctly aligned reads w.r.t. the read distance (top), and read length in correctly aligned reads (bottom), on Chr22 (left), Chr1 (center) and AllChr (right), for real PacBio CLR read sets. Plots for minigraph and minichain can be found in the Supplementary Data

***Real reads on chromosome and whole human genome graphs.*** In this case, the difference between GraphAligner and GraphChainer is even clearer (Fig. 3). Since these graphs are larger, GraphAligner’s seeds are more likely to have false occurrences in the graph, and thus extending each seed (cluster) *individually* leads to worse alignments in more cases. As shown in Table 2, for criterion σ=0.3, GraphChainer’s improvements in correctly aligned reads, and in total length of correctly aligned reads, are up to 17.02%, and 28.68%, respectively, for Chr22. For Chr1, the improvement in the two metrics is smaller, up to 12.74%, and 21.80%, but still significant. For AllChr, the relative improvement in the metrics rises up to 16.52% and 26.40%. Both aligners decrease in accuracy when considering the *whole* human genome variation graph, but GraphChainer is more resilient to this change. (Recall that real reads used for AllChr are a uniform random sample of real reads used for Chr1.) We also note that GraphChainer reaches an accuracy of 95% for σ<0.3, whereas this happens at σ>0.5 for GraphAligner.

**Table 2.** Correctly aligned reads with respect to the distance, and percentage of read length in correctly aligned reads, for $\sigma_{\mathrm{read}}=0.3$ (i.e. the edit distance between the read and the reported sequence can be up to 30% of the read length) for real PacBio CLR read sets.^a^

Graph	Aligner	Correctly aligned	Good length
Chr22	GraphChainer	96.48% (+17.02%)	96.30% (+28.68%)
(real)	–Step 2	95.31% (+15.59%)	95.01% (+26.95%)
	–Step 3	94.34% (+14.41%)	93.82% (+25.36%)
	GraphAligner	82.45%	74.84%
	minigraph	22.38%	33.25%
	minichain	1.52%	1.95%
Chr1	GraphChainer	95.01% (+12.74%)	94.63% (+21.80%)
(real)	–Step 2	93.12% (+10.50%)	92.08% (+18.51%)
	–Step 3	91.75% (+8.87%)	89.95% (+15.77%)
	GraphAligner	84.27%	77.69%
	minigraph	25.95%	38.40%
	minichain	0.84%	1.02%
AllChr	GraphChainer	84.02% (+16.52%)	86.75% (+26.40%)
(real)	–Step 2	82.73% (+14.73%)	85.05% (+23.93%)
	–Step 3	81.69% (+13.29%)	83.53% (+21.62%)
	GraphAligner	72.11%	68.63%
	minigraph	19.42%	28.47%
	minichain	6.37%	7.10%

a Percentages in parentheses are relative improvements w.r.t. GraphAligner.

#### 3.3.2 Results of minigraph and minichain

For both simulated reads with 15% error rate and real reads on all variation graphs presented in the main text minigraph and minichain align <60% of reads for all criteria. (For presentation reasons we keep these results in the tables but exclude them from the plots. The corresponding plots can be found in Supplementary Figs S7, S8, S11, S12, S15, S16, S19, S20, S23, and S24.) In the case of simulated reads, at the weaker overlap criterion of δ=0.1, minigraph correctly aligns close to 30% of reads in all graphs except Chr22 (with an accuracy of ∼50%), and minichain drops to ∼10% (except Chr22 with ∼30% accuracy). In the case of real reads, and for criterion $\sigma_{\mathrm{read}}=0.3$, minigraph correctly aligns <30% of reads, but this significantly (∼10%) increases when considering the total read length, whereas minichain drops in accuracy to <2%.

The main reason on the low accuracy of minigraph and minichain is that these tools do not work well on the highly variable variation graphs tested (graphs with lots of small variants such as SNPs), as reported at https://github.com/lh3/minigraph#limitations, which is intensified with the high error rate of 15% on reads simulated with Badread (Wick 2019) and the high error rate in our real PacBio CLR reads. Results on simulated reads with lower 5% error rate (these can be found Supplementary Tables S5, S10, S12, and S14 and Figs S26–40) show a significant improvement in these tools with minigraph obtaining accuracy around 90% and minichain around 60% for δ=0.1. Moreover, at the same error rate and δ=0.1, and for the (less variable) graphs 10H and 95H tested on minichain’s publication, minigraph achieves accuracy of 93.59% and minichain of 94.80% for 10H, and 81.94% and 83.97% for 95H, beating both GraphAligner and GraphChainer.

***Performance.***  Supplementary Table S6 shows the running time and peak memory of all aligners on real reads and Supplementary Table S8 on simulated reads with 15% error rate. Even though GraphChainer takes ∼2–8× time and ∼1.5–2.5× memory resources compared to GraphAligner, these requirements are still within the capabilities of a modern high-performance computer. Running times are larger on real reads on Chr1 and AllChr due to its bigger size, but also to its larger read coverage in the case of Chr1. To explore other tradeoffs between memory and time performance and accuracy, we ran GraphChainer with step in {1, 2, 3}. We observe that the alignment accuracy remains significantly above GraphAligner’s, while the running time shrinks by up to half: this is the case of Chr1 and AllChr where the accuracy of step  =3 is still an 8% (91.75% versus 84.27%) and 13% (81.69% versus 72.11%) improvement over GraphAligner, respectively. GraphAligner and minigraph use the less memory and are the fastest. minigraph slightly outperforms GraphAligner in both metrics due to the minimizer index built by GraphAligner. minichain has a similar indexing time and peak memory as GraphChainer, since they are both variations of the same algorithm (Mäkinen *et al.* 2019). minichain has the fastest alignment time after indexing, which can be explained by the low accuracy obtained by the aligner. Supplementary Table S7 shows that one bottleneck of GraphChainer is obtaining the anchors.

## 4 Conclusions

The pangenomic era has given rise to several methods, including the vg toolkit (Garrison *et al.* 2018), for accurately and efficiently aligning short reads to variation graphs. However, these tools fail to scale when considering the more-erroneous long reads and much less work has been published around this problem. GraphAligner (Rautiainen and Marschall 2020) is the state-of-the-art for aligning long reads to (whole human genome) variation graphs. Here, we have presented the first efficient implementation of co-linear chaining on a string labeled DAG when allowing one-node suffix–prefix overlaps. We showed that our new method, GraphChainer, significantly improves the alignments of GraphAligner, on real PacBio CLR reads and whole human genome variation graphs. We showed that co-linear chaining, successfully used by sequence-to-sequence aligners (Li 2018), is a useful technique also in the context of variation graphs.

One of the main drawbacks of our formulation of co-linear chaining is that the optimization criterion used by GraphChainer, the coverage of the read, is blind to gaps between anchor paths in the variation graph. Considering such gaps in both objects has resulted in interesting connections between co-linear chaining and classical distance metrics in the linear case (Mäkinen and Sahlin 2020, Jain *et al.* 2022). The recent works of minigraph and minichain have considered such gaps, showing an increase in accuracy but still relying on heuristics to approximate the optimum value of the chaining.

## Supplementary Material

btad460_Supplementary_Data
