# Supplementary material for: Chaining for accurate alignment of erroneous long reads to acyclic variation graphs
Source: Bioinformatics. 2023 Jul 26;39(8):btad460. doi: 10.1093/bioinformatics/btad460 (PMC10423031; doi:10.1093/bioinformatics/btad460)
Supplement: btad460_Supplementary_Data [file btad460_supplementary_data.pdf]

1 Statistics on variation graphs

Table 3. Statistics of every dataset used in our experiments. Every graph has a read set simulated on a random path of the graph. Graphs Chr22, Chr1 and AllChr were built using GRCh37 as the reference and variants from the 1000 Genomes Project, these graphs have an additional PacBio CLR read set (rows "real") obtained from SRA. Column k is the width of the graph. In the case of 10H, 95H and AllChr, k corresponds to the maximum width of a connected component (chromosome graph).

Graph	#Nodes	Labels bps	k	#Reads	Tot. reads bps	Cov
LRC	117 787	1 099 856	4	1 093	15 872 214	15×
MHC1	479 531	5 138 362	4	5 091	74 524 274	15×
Chr22	3 197 160	52 423 213	7	52 464	769 238 818	15×
real				136 494	2 858 621 416	56×
Chr1	18 807 963	255 754 179	9	254 251	3 736 803 386	15×
real				907 572	19 617 046 919	79×
AllChr	238 097 524	3 180 963 894	9	202 062	2 968 612 605	1×
real				143 697	3 095 684 582	1×
10H	141 755	3 153 388 445	9	67 830	991 320 129	0.3×
95H	611 949	3 335 591 586	35	51 898	761 788 975	0.2×

Table 4. Statistics of variation graphs of human chromosomes built with the `vg` toolkit using GRCh37 as the reference, and variants from the 1000 Genomes Project phase 3 release (Clarke et al., 2016). The number of nodes is from the compact representation where a non-branching path is merged into a single node. Each graph is a DAG, and the numbers refer only to one of the two weakly connected components (one being the reverse complement of the other). Intuitively, the width of a graph is a measure of the variability of its most complex zone, since `vg` will iteratively split those zones when building the graph. It is worth noting that these graphs are acyclic excluding structural variants.

Chr	#Nodes	Labels bps	Width
1	18 807 963	255 754 179	9
2	20 597 735	250 312 064	6
3	16 965 471	203 883 122	7
4	16 662 965	196 912 161	6
5	15 313 396	186 204 491	6
6	14 596 952	176 169 819	9
7	13 707 868	163 880 288	8
8	13 370 501	150 986 669	8
9	10 355 761	144 794 206	7
10	11 595 921	139 553 125	6
11	11 760 609	139 076 341	7
12	11 239 568	137 745 335	6
13	8 304 603	118 040 865	6
14	7 714 611	110 019 784	7
15	7 045 787	104 968 212	6
16	7 837 615	93 074 017	8
17	6 758 004	83 545 050	6
18	6 592 253	80 357 608	7
19	5 306 144	60 981 512	6
20	5 268 137	64 854 544	6
21	3 207 166	49 243 683	6
22	3 197 160	52 423 213	7
X	10 011 934	158 748 581	9
Y	184 003	59 435 025	2

2 Co-linear chaining in time $O(kN \log kN)$

As we discussed, the pseudocode of Algorithm 1 takes $O(kN)$ updates and queries to the data structures, which can be answered in $O(\log N)$ time each, thus adding up to $O(kN \log N)$ time in total. However, the for loops iterate over all the vertices of the graph, and over all the forward links, adding an $O(k|V|)$ additional time to the chaining process.

Having such $O(k|V|)$ time while chaining does not change the $O(k(|V| + |E|) \log |V| + kN \log N)$ asymptotic running time of our algorithm, but it affects the practical performance of chaining. As such, we decided to divide the algorithm into pre-processing the DAG, that we solve in $O(k^3|V| + k|E|)$ time by using a recent result to compute a minimum path cover of a DAG (Cáceres *et al.*, 2022), and chaining that we solve in time $O(kN \log kN)$[19].

To remove the chaining's dependency on the graph size we get rid of the for loops of Algorithm 1 by instead processing the anchors (plus the required objects) in an order that simulates the for loop order of Algorithm 1. In the case of **Step 1**, this order is simulated by sorting the anchors by topological order of the path endpoints[20], which can be done in $O(N \log N)$ time (by previously computing the topological ordering on pre-processing time). For **Step 0**, we also require to sort the anchors by the topological order of the path starting points, as well as interleaving the computation of **Step 0.1** and **Step 0.2**. For interleaving the computation of **Step 0.1** and **Step 0.2** (as well as the computation of **Step 0**, **Step 1** and **Step 2**), we partition the corresponding sorted arrays, such that each part corresponds to a different vertex, which can be done in extra $O(N)$ time ($O(kN)$ time in the case of **Step 2**). To interleave the computation of the different steps, we process the sorted arrays by parts according to the topological ordering of the corresponding paths. Finally, to simulate the for loop of **Step 2**, we sort the tuples (s, P_i, A_j) (such that $(A_j.s, P_i) \in \texttt{forward[s]}$) by topological order of s. Since there can be $O(kN)$ such tuples, this takes $O(kN \log kN)$ time.

Algorithm 2 shows an alternative implementation of these ideas, which is simpler to write. Iterations of every step are encoded in 5-tuples, which are sorted to simulate the processing order of Algorithm 1. Note that $|S| = O(kN)$, thus the sorting runs in time $O(kN \log kN)$, which dominates the running time of the algorithm. The first coordinate of the tuple is a vertex, which simulates the outer for-loop of Algorithm 1. The second coordinate is $A_j.o_s$ and $A_j.o_t$ for **Step 0.1** and **Step 0.2** and ∞ for **Step 1** and **Step 2** to simulate the for-loop of **Step 0**. The third coordinate corresponds to the `step` number and sets the relative order between the different steps. Finally, the last two coordinates are present to recover the corresponding anchor and path to process.

3 CLC in general graphs

We will show that a slight modification of our solution for Problem 2 allows us to efficiently solve an analogous definition of co-linear chaining for cyclic graphs. We will give a formal definition of this problem, show and prove how to solve it, and discuss the limitations of this approach.

In this (more general) case we allow G to contain directed cycles. We also allow the anchor paths to repeat vertices, and thus we change the notation $A.P$ by $A.W$, where $A.W$ is the anchor *walk* of A. We maintain the optimization criterion, but slightly change the definition of precedence between anchor paths to handle cyclic graphs.

[19] Note that we worsen running time by a k factor inside the log factor. However, $k \ll N$, thus the asymptotic running time is maintained.

[20] If there are ties, they are sorted by endpoint in the string label of the path endpoint. This is done in this case as well as the other sortings, but we remove it from the phrasing by ease of explanation.

Algorithm 2: $O(kN \log kN)$ time version of Algorithm 1. A topological order on the vertices of G is assumed. In line 5, the 5-tuples used by the algorithm are sorted lexicographically, that is, sorting according to the first coordinate (in this case by topological order) and in case of ties sorting according to the next coordinates.

Input : A path cover $\mathcal{P} = P_1, \ldots, P_k$ of G, anchors $\mathcal{A} = A_1, \ldots, A_N$.
Output: A chain $\mathcal{C}$ of maximum coverage.

1 $S_{0.1} \leftarrow \{(A_j.s, A_j.o_s, 0.1, 0, j) \mid A_j \in \mathcal{A}\}$
2 $S_{0.2} \leftarrow \{(A_j.t, A_j.o_t, 0.2, 0, j) \mid A_j \in \mathcal{A}\}$
3 $S_1 \leftarrow \{(A_j.t, \infty, 1, i, j) \mid A_j.t \in P_i\}$
4 $S_2 \leftarrow \{(v, \infty, 2, i, j) \mid (A_j.s, P_i) \in$ `forward[v]`$\}$
5 $S \leftarrow$ `sort`$(S_{0.1} \cup S_{0.2} \cup S_1 \cup S_2)$
6 `initialize`$(\mathcal{D}_i^{\not\nearrow})$ for every $i \in \{1, \ldots, k\}$
7 `new_step_0` $\leftarrow$ `True`
8 **for** $(v, p, step, i, j) \in S$ **do**
9   **if** $step < 1$ **then**
10   **if** *new_step_0* **then**
11   `initialize`$(\mathcal{U}^{\not\nearrow})$
12   `new_step_0` $\leftarrow$ `False`
13   **if** $step = 0.1$ **then**
14   `C[j]` $\leftarrow$ max(`C[j]`, $A_j.y - A_j.x + 1 + \mathcal{U}^{\not\nearrow}$.`rMq`$(0, A_j.x - 1)$)
15   **if** $step = 0.2$ **then**
16       $\mathcal{U}^{\not\nearrow}$.`update`$(A_j.y,$ `C[j]`$)$
17   **else**
18   `new_step_0` $\leftarrow$ `True`
19   **if** $step = 1$ **then**
20       $\mathcal{D}_i^{\not\nearrow}$.`update`$(A_j.y,$ `C[j]`$)$
21   **if** $step = 2$ **then**
22   `C[j]` $\leftarrow$ max(`C[j]`, $A_j.y - A_j.x + 1 + \mathcal{D}_i^{\not\nearrow}$.`rMq`$(0, A_j.x - 1)$)
23 **return** Maximum coverage chain $\mathcal{C}$ recovered from `C`

Problem 3 (CLC in general graphs). *Given a* string labeled *graph* $G = (V, E)$ *and a set* $\mathcal{A} = \{A_1, \ldots, A_N\}$ *of anchors, find a chain* $\mathcal{C} = A_{i_1}, \ldots, A_{i_q}$ *maximizing* $cov(\mathcal{C}) := |\bigcup_{j=1}^{q} A_{i_j}.I|$, *such that for all* $j \in \{1, \ldots, q-1\}$, A_{i_j} precedes $A_{i_{j+1}}$, *meaning* $A_{i_j}.y < A_{i_{j+1}}.x$ *and* $A_{i_j}.t$ *reaches* $A_{i_{j+1}}.s$, *but if* $A_{i_j}.t = A_{i_{j+1}}.s$ *and* $A_{i_{j+1}}.s$ *does not strictly reaches itself we also require that* $A_{i_j}.o_t < A_{i_{j+1}}.o_s$.

Note that the change in path precedence allows to connect paths with one-node suffix prefix overlap even when their respective offsets are inverted, but only in the case when the overlap can (strictly) reach itself. Also note that Problem 3 is a generalization of Problem 2, since in the DAG case whenever $A_{i_j}.t = A_{i_{j+1}}.s$ it follows that $A_{i_{j+1}}.s$ does not strictly reaches itself (otherwise we would obtain a cycle).

We solve Problem 3 by using a slight modification of our algorithm for Problem 2 in the condensation of G. The *condensation* $\mathcal{G} = (\mathcal{V}, \mathcal{E})$ of a graph $G = (V, E)$ is the DAG of its strongly connected components, which can be computed in $O(|V| + |E|)$ time (Tarjan, 1972; Dijkstra, 1976; Sharir, 1981). More precisely, $\mathcal{V} = \{S \mid S \text{ is a strongly connected component of } G\}$, and $\mathcal{E} = \{(S, S') \mid \exists u \in S, v \in S', (u, v) \in E\}$. Since the strongly connected components of a graph form a partition of the vertices, every vertex of G can be mapped to its corresponding vertex (component) in $\mathcal{G}$. As such, a walk in G can be mapped to its corresponding path in $\mathcal{G}$ (the ordered sequence of components it visits in the walk). Therefore, any path cover of G can be mapped into a path cover of $\mathcal{G}$, thus $\mathsf{width}(G) = k \geq k' = \mathsf{width}(\mathcal{G})$. To finish our reduction we map the input anchors $\mathcal{A}$ to $\mathcal{A}'$ by mapping the corresponding anchor walks to paths in $\mathcal{G}$. Let us call this anchor transformation $f_{\mathcal{G}}$, such that, $f_{\mathcal{G}}(A) = (A.I, P)$, where P is the corresponding path of $A.W$ in $\mathcal{G}$, and thus $\mathcal{A}' = f_{\mathcal{G}}(\mathcal{A})$. Note that this transformation is incomplete as it does not specify the offsets $f_{\mathcal{G}}(A).o_s, f_{\mathcal{G}}(A).o_t$.

If $f_{\mathcal{G}}(A).s$ (resp. $f_{\mathcal{G}}(A).t$) only contains $A.s$ (resp. $A.t$) then it makes sense to use $A.o_s$ ($A.o_t$). However, if $f_{\mathcal{G}}(A).s$ ($f_{\mathcal{G}}(A).t$) contains other vertices then $f_{\mathcal{G}}(A).o_s$ ($f_{\mathcal{G}}(A).o_t$) is undefined since $f_{\mathcal{G}}(A).s$ ($f_{\mathcal{G}}(A).t$) is not a linear structure anymore, but a subgraph. The following lemma proves that in this case it is not necessary to compute $f_{\mathcal{G}}(A).o_s$ ($f_{\mathcal{G}}(A).o_t$).

Lemma 1. *Following the precedence definition of Problem 3. If* $|f_{\mathcal{G}}(A).t| > 1$ *or* $|f_{\mathcal{G}}(A').s| > 1$, *then* A *precedes* A' *if and only if* $A.y < A'.y$ *and* $A.t$ *reaches* $A'.s$.

Proof. The forward direction of the equivalence follows by the precedence definition. The backwards direction also follows by definition if $A.t \neq A'.s$. Otherwise if $A.t = A'.s$, note that $A'.s$ strictly reaches itself, since $|f_{\mathcal{G}}(A).t| = |f_{\mathcal{G}}(A').s| > 1$.

Algorithm 3: Our solution to Problem 3. For a strongly connected component u, the entry `forward[u]` contains the pairs (v, P_i), such that u is the last strongly connected component, in path P_i, that reaches v. These links can be pre-computed in time $O(k'|E|)$ on the condensation $\mathcal{G}$ of G, and there are $O(k'|V|)$ of them in total (Mäkinen *et al.*, 2019). Data structures $\mathcal{U}^{\not\nearrow}$ and $\mathcal{D}_i^{\not\nearrow}, i \in \{1, \ldots, k'\}$ can answer `update` and `rMq`. We assume that the condensation $\mathcal{G} = (\mathcal{V}, \mathcal{E})$ of G is already computed, as well as the mapping of anchors $f_{\mathcal{G}}$. We use *inc.* to denote that the preceding set is scanned in increasing order.

Input : A path cover $\mathcal{P} = P_1, \ldots, P_{k'}$ of $\mathcal{G}$, anchors $\mathcal{A} = A_1, \ldots, A_N$.
Output: A chain $\mathcal{C}$ of maximum coverage.

`initialize`$(\mathcal{D}_i^{\not\nearrow})$ for every $i \in \{1, \ldots, k'\}$
for $v \in \mathcal{V}$ *in topological order* **do**
  `initialize`$(\mathcal{U}^{\not\nearrow})$
  if $|v| = 1$ **then**
    for $o \in \{A_j.o_s \mid A_j.s = v\} \cup \{A_j.o_t \mid A_j.t = v\}$ *inc.* **do**
      for A_j *with* $A_j.o_s = o \wedge A_j.s = v$ **do**
        `C[j]` $\leftarrow \max($`C[j]`$, A_j.y - A_j.x + 1 + \mathcal{U}^{\not\nearrow}.$`rMq`$(0, A_j.x - 1))$
      for A_j *with* $A_j.o_t = o \wedge A_j.t = v$ **do**
        $\mathcal{U}^{\not\nearrow}.$`update`$(A_j.y,$ `C[j]`$)$
  else
    for $y \in \{A_j.y \mid A_j.s = v \vee A_j.t = v\}$ *inc.* **do**
      for A_j *with* $A_j.y = y \wedge A_j.s = v$ **do**
        `C[j]` $\leftarrow \max($`C[j]`$, A_j.y - A_j.x + 1 + \mathcal{U}^{\not\nearrow}.$`rMq`$(0, A_j.x - 1))$
      for A_j *with* $A_j.y = y \wedge A_j.t = v$ **do**
        $\mathcal{U}^{\not\nearrow}.$`update`$(A_j.y,$ `C[j]`$)$
  for A_j *with* $A_j.t = v$ **do**
    for $P_i \in \{P \in \mathcal{P} \mid v \in P\}$ **do**
      $\mathcal{D}_i^{\not\nearrow}.$`update`$(A_j.y,$ `C[j]`$)$
  for $(w, P_i) \in$ `forward[v]` **do**
    for A_j *with* $A_j.s = w$ **do**
      `C[j]` $\leftarrow \max($`C[j]`$, A_j.y - A_j.x + 1 + \mathcal{D}_i^{\not\nearrow}.$`rMq`$(0, A_j.x - 1))$
return Maximum coverage chain $\mathcal{C}$ recovered from `C`

Therefore, we will proceed as in Algorithm 1 when $|f_{\mathcal{G}}(A).s| = 1$ or $|f_{\mathcal{G}}(A).t| = 1$, and otherwise we will run **Step 0** by increasing value of interval endpoint. Algorithm 3 shows the pseudocode for our solution, which runs in time $O(|V| + |E| + N + k'(|\mathcal{V}| + |\mathcal{E}|) \log |\mathcal{V}| + k'N \log N) = O(k(|V| + |E|) \log |V| + kN \log N)$.

4 Commands for running the tools

Our code, datasets and pipeline can be found at:
https://github.com/algbio/GraphChainer

Read simulation with BadReads:

```
badread simulate --seed {seed} --reference {Ref}
--quantity 15x --length 15000,10000
--error_model pacbio2016 --identity 85,95,5
```

Variation graph construction with `vg` toolkit (same parameters as those used for `GraphAligner`'s experiments (Rautiainen and Marschall, 2020, p.24)):

```
vg construct -t 30 -a -r {ref} -v {vcf} -R {chr}
-p -m 3000000
```

AllChr was built by taking the union of the 24 chromosome graphs.

Read alignment (same parameters for `GraphAligner` as used on its variation graph experiments (Rautiainen and Marschall, 2020, p.24), default parameters for `PaSGAL` (Jain *et al.*, 2019), same parameters for the extension of `AStarix` on its experiments for long reads (Ivanov *et al.*, 2021, p.13)). For `minigraph` and `minichain` we used the default parameters with the recommended option `c`:

```
GraphAligner -t {Threads} -x vg -f {Reads} -g {Graph}
-a {long_gam}

GraphChainer -t {Threads} -f {Reads} -g {Graph}
-a {clc_gam}

minigraph -t {Threads} -c {Graph} {Reads}
> {minigraph_gaf}

minichain -t {Threads} -c {Graph} {Reads}
> {minichain_gaf}

PaSGAL -m vg -r {Graph} -q {Reads} -t {Threads}
-o {output_file}

astarix align-optimal -a astar-seeds -g {Graph}
-q {Reads} --fixed_trie_depth 1 --seeds_len 150
-D 14 -G 1 -S 1
```

5 Further experimental results

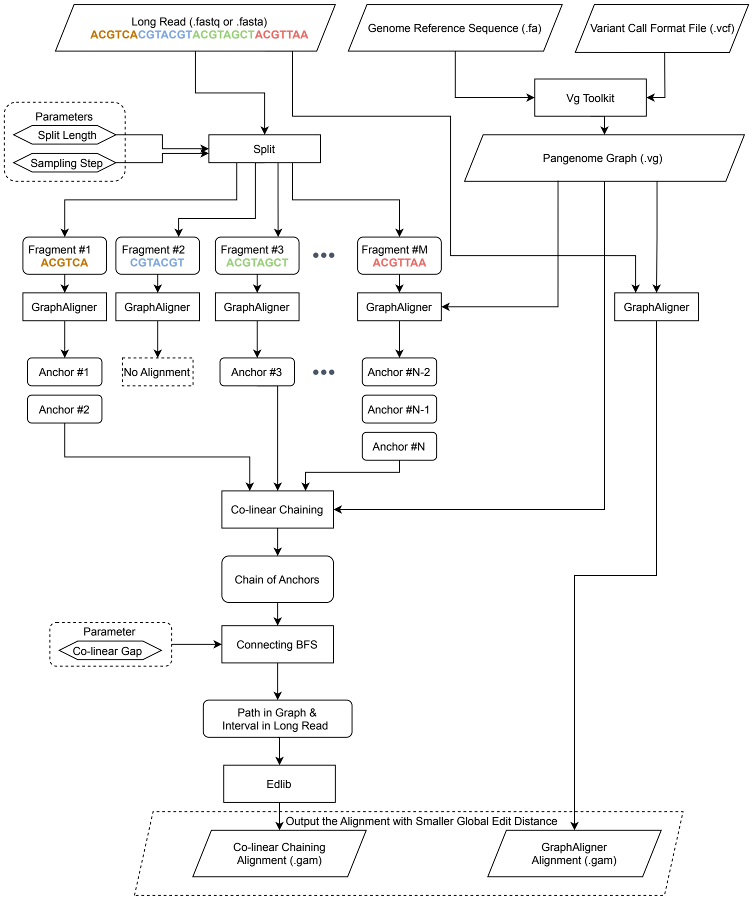

Fig. 4: The flow diagram of `GraphChainer`: a long-read is split into fragments (of default length 35), which are aligned with `GraphAligner` (though any other method to extract anchors can be used). Anchors are created for each alignment of a fragment. The anchors are chained with our algorithm from Section 2.3. The optimal chain is split whenever the BFS shortest path between consecutive anchor paths is longer than a co-linear gap limit parameter (default 10 000), and the longest resulting path is kept. If the edit distance between the long read and this path is better than the one obtained by aligning the entire long read with `GraphAligner`, then this alignment is output; otherwise `GraphAligner`'s alignment is reported.

Table 5. Correctly aligned reads with respect to the overlap for $\delta \in \{0.1, 0.85\}$ (i.e., the overlap between the reported path and the ground truth is at least 10% or 85% of the length of the ground truth sequence, respectively) for the simulated read sets with error rate 5% as opposed to 15% shown in Table 1. Percentages in parentheses are relative improvements w.r.t. `GraphAligner`. 10H and 95H are the smallest and largest graphs used in the experiments of `minichain`, respectively.

Graph	Aligner	Correctly aligned	
		$\delta = 0.1$	$\delta = 0.85$
LRC	GraphChainer	98.90% (+0.65%)	98.72% (+2.57%)
	GraphAligner	98.26%	96.24%
	minigraph	85.60%	67.71%
	minichain	45.78%	9.72%
MHC1	GraphChainer	99.64% (+0.42%)	99.52% (+2.53%)
	GraphAligner	99.23%	97.07%
	minigraph	88.57%	71.88%
	minichain	53.60%	17.88%
Chr22	GraphChainer	99.62% (+0.50%)	99.45% (+2.24%)
	GraphAligner	99.12%	97.28%
	minigraph	91.12%	79.88%
	minichain	66.49%	41.64%
Chr1	GraphChainer	99.41% (+0.46%)	99.06% (+2.33%)
	GraphAligner	98.96%	96.81%
	minigraph	89.30%	75.45%
	minichain	60.81%	25.87%
AllChr	GraphChainer	99.70% (+0.47%)	99.36% (+2.78%)
	GraphAligner	99.23%	96.67%
	minigraph	87.20%	67.87%
	minichain	53.28%	16.04%
10H	GraphChainer	95.79% (+0.53%)	95.48% (+2.38%)
	GraphAligner	95.28%	93.27%
	minigraph	93.59%	89.72%
	minichain	94.80%	92.18%
95H	GraphChainer	80.48% (+0.98%)	79.90% (+2.63%)
	GraphAligner	79.70%	77.85%
	minigraph	81.94%	73.59%
	minichain	83.97%	77.88%

Table 6. Wall-clock running time (in format hh:mm:ss) and peak memory (Mem, in GBs) when aligning real PacBio CLR read sets. Column "Map time" corresponds to the "Total time" minus indexing time.

Graph	Aligner	Mem	Total time	Map time
Chr22 (real)	GraphChainer	8.11	00:27:15	00:26:50
	-step 2	7.97	00:21:59	00:21:34
	-step 3	8.03	00:20:17	00:19:52
	GraphAligner	4.86	00:11:34	00:11:17
	minigraph	5.59	00:41:59	00:41:53
	minichain	8.13	00:04:28	00:03:26
Chr1 (real)	GraphChainer	38.65	06:39:55	06:37:25
	-step 2	38.71	04:18:01	04:15:31
	-step 3	38.76	03:31:59	03:29:29
	GraphAligner	15.99	01:19:29	01:17:57
	minigraph	12.77	05:04:27	05:03:54
	minichain	31.42	00:57:11	00:48:18
AllChr (real)	GraphChainer	419.33	10:09:55	08:12:24
	-step 2	419.64	06:22:10	04:24:39
	-step 3	421.31	05:08:53	03:11:22
	GraphAligner	183.74	01:28:04	00:24:52
	minigraph	117.53	00:53:34	00:45:15
	minichain	394.64	02:27:46	00:12:26

Table 7. CPU time spent in the main phases of `GraphChainer` on Chr22, Chr1 and AllChr (in format hh:mm:ss) on the real PacBio CLR reads. The column `edlib` is the time needed to run `edlib` between the read and the path found by co-linear chaining to finally report the best solution.

Graph	step	Getting anchors	Co-linear chaining	edlib
Chr22	1	01:19:58	00:25:05	00:46:35
Chr22	2	00:39:48	00:10:56	00:48:10
Chr22	3	00:26:18	00:06:22	00:49:14
Chr1	1	30:29:40	15:12:30	05:39:41
Chr1	2	15:15:18	06:38:22	05:50:56
Chr1	3	10:08:13	04:03:18	05:54:59
AllChr	1	60:50:41	14:47:32	01:10:04
AllChr	2	28:23:53	06:44:49	01:07:54
AllChr	3	20:16:38	04:07:24	01:06:12

Table 8. Wall-clock running time (in format hh:mm:ss) and peak memory (Mem, in GBs) when aligning simulated reads with 15% error rate. Column "Map time" corresponds to the "Total time" minus the time taken on indexing for the respective tool.

Graph	Aligner	Mem	Total time	Map time
LRC	GraphChainer	0.86	00:00:06	00:00:05
	GraphAligner	0.69	00:00:04	00:00:03
	minigraph	1.11	00:00:06	00:00:05
	minichain	0.66	00:00:01	00:00:01
MHC1	GraphChainer	1.71	00:00:28	00:00:24
	GraphAligner	1.21	00:00:18	00:00:16
	minigraph	1.75	00:00:30	00:00:29
	minichain	1.58	00:00:07	00:00:03
Chr22	GraphChainer	7.38	00:05:25	00:05:00
	GraphAligner	4.00	00:02:46	00:02:29
	minigraph	4.89	00:03:32	00:03:26
	minichain	7.58	00:01:54	00:00:52
Chr1	GraphChainer	38.49	01:04:07	01:01:37
	GraphAligner	15.75	00:14:36	00:13:04
	minigraph	12.35	00:21:37	00:21:04
	minichain	30.69	00:13:57	00:05:04
AllChr	GraphChainer	417.47	10:02:08	08:04:37
	GraphAligner	183.73	01:28:24	00:25:12
	minigraph	117.21	00:23:14	00:14:55
	minichain	394.27	02:23:41	00:08:12
10H	GraphChainer	87.91	03:02:58	02:54:27
	GraphAligner	58.35	00:12:46	00:06:02
	minigraph	23.17	00:02:49	00:01:53
	minichain	25.54	00:06:14	00:05:18
95H	GraphChainer	141.00	03:14:38	03:03:13
	GraphAligner	55.85	00:12:14	00:05:08
	minigraph	24.74	00:02:59	00:01:58
	minichain	24.65	00:12:40	00:11:34

Table 9. Read length in correctly aligned reads with respect to the overlap for $\delta \in \{0.1, 0.85\}$ (i.e., the overlap between the reported path and the ground truth is at least 10% or 85% of the length of the ground truth sequence, respectively) for the simulated read sets. Percentages in parentheses are relative improvements w.r.t. `GraphAligner`.

Graph	Aligner	Good length	
		$\delta = 0.1$	$\delta = 0.85$
LRC	`GraphChainer`	99.52% (+4.02%)	98.18% (+8.37%)
	`GraphAligner`	95.67%	90.60%
	`minigraph`	34.26%	8.46%
	`minichain`	7.51%	0.52%
MHC1	`GraphChainer`	99.50% (+2.79%)	98.44% (+7.36%)
	`GraphAligner`	96.80%	91.70%
	`minigraph`	36.71%	14.21%
	`minichain`	3.60%	0.47%
Chr22	`GraphChainer`	99.46% (+3.33%)	98.19% (+6.83%)
	`GraphAligner`	96.26%	91.91%
	`minigraph`	53.16%	34.32%
	`minichain`	32.41%	28.17%
Chr1	`GraphChainer`	99.17% (+3.30%)	96.73% (+5.62%)
	`GraphAligner`	96.00%	91.58%
	`minigraph`	42.14%	19.36%
	`minichain`	11.73%	8.70%
AllChr	`GraphChainer`	99.30% (+3.53%)	96.59% (+6.15%)
	`GraphAligner`	95.91%	90.99%
	`minigraph`	32.20%	9.98%
	`minichain`	2.74%	1.49%

Table 10. Read length in correctly aligned reads with respect to the overlap for $\delta \in \{0.1, 0.85\}$ (i.e., the overlap between the reported path and the ground truth is at least 10% or 85% of the length of the ground truth sequence, respectively) for the simulated read sets with error rate 5% as opposed to 15% shown in Table 9. Percentages in parentheses are relative improvements w.r.t. `GraphAligner`. 10H and 95H are the smallest and largest graphs used in the experiments of `minichain`, respectively.

Graph	Aligner	Good length	
		$\delta = 0.1$	$\delta = 0.85$
LRC	`GraphChainer`	99.37% (+0.81%)	98.92% (+3.57%)
	`GraphAligner`	98.57%	95.51%
	`minigraph`	88.54%	76.07%
	`minichain`	55.79%	12.98%
MHC1	`GraphChainer`	99.83% (+0.54%)	99.69% (+3.64%)
	`GraphAligner`	99.30%	96.19%
	`minigraph`	90.80%	78.77%
	`minichain`	61.38%	25.20%
Chr22	`GraphChainer`	99.79% (+0.51%)	99.61% (+2.97%)
	`GraphAligner`	99.28%	96.73%
	`minigraph`	92.75%	84.82%
	`minichain`	72.78%	46.30%
Chr1	`GraphChainer`	99.60% (+0.45%)	99.20% (+3.17%)
	`GraphAligner`	99.15%	96.14%
	`minigraph`	91.21%	81.29%
	`minichain`	68.23%	32.94%
AllChr	`GraphChainer`	99.81% (+0.49%)	99.44% (+3.84%)
	`GraphAligner`	99.33%	95.77%
	`minigraph`	89.68%	75.16%
	`minichain`	61.71%	22.91%
10H	`GraphChainer`	95.15% (+0.54%)	94.75% (+3.06%)
	`GraphAligner`	94.64%	91.94%
	`minigraph`	93.52%	90.18%
	`minichain`	94.71%	92.52%
95H	`GraphChainer`	78.08% (+1.06%)	77.38% (+3.36%)
	`GraphAligner`	77.26%	74.87%
	`minigraph`	81.52%	74.08%
	`minichain`	83.26%	78.01%

Table 11. Correctly aligned reads with respect to the distance, for $\sigma_{truth} = 0.3$ (i.e., the edit distance between the truth sequence and the reported sequence can be up to 30% of the truth sequence length), and $\sigma_{read} = 0.3$ (i.e., the edit distance between the read and the reported sequence can be up to 30% of the read length) for simulated read sets. Percentages in parentheses are relative improvements w.r.t. `GraphAligner`.

Graph	Aligner	Correctly aligned	
		$\sigma_{truth} = 0.3$	$\sigma_{read} = 0.3$
LRC	`GraphChainer`	98.72% (+5.78%)	96.25% (+5.31%)
	`GraphAligner`	93.32%	91.40%
	`minigraph`	13.36%	11.44%
	`minichain`	1.56%	1.28%
MHC1	`GraphChainer`	98.82% (+5.10%)	96.64 (+4.22%)
	`GraphAligner`	94.03%	92.73%
	`minigraph`	17.13%	14.04%
	`minichain`	0.73%	0.55%
Chr22	`GraphChainer`	98.86% (+4.93%)	96.43% (+4.06%)
	`GraphAligner`	94.22%	92.67%
	`minigraph`	36.77%	33.33%
	`minichain`	28.59%	26.67%
Chr1	`GraphChainer`	98.19% (+4.41%)	95.73% (+3.42%)
	`GraphAligner`	94.04%	92.57%
	`minigraph`	22.66%	19.36%
	`minichain`	9.07%	8.32%
AllChr	`GraphChainer`	97.82% (+4.78%)	95.33% (+3.73%)
	`GraphAligner`	93.36%	91.90%
	`minigraph`	12.86%	10.46%
	`minichain`	1.54%	1.42%

Table 12. Correctly aligned reads with respect to the distance, for $\sigma_{truth} = 0.3$ (i.e., the edit distance between the truth sequence and the reported sequence can be up to 30% of the truth sequence length), and $\sigma_{read} = 0.3$ (i.e., the edit distance between the read and the reported sequence can be up to 30% of the read length) for simulated read sets with error rate 5% as opposed to 15% shown in Table 11. Percentages in parentheses are relative improvements w.r.t. `GraphAligner`. 10H and 95H are the smallest and largest graphs used in the experiments of `minichain`, respectively.

Graph	Aligner	Correctly aligned	
		$\sigma_{truth} = 0.3$	$\sigma_{read} = 0.3$
LRC	`GraphChainer`	99.72% (+1.78%)	99.45% (+1.69%)
	`GraphAligner`	97.98%	97.80%
	`minigraph`	77.16%	75.50%
	`minichain`	19.91%	18.99%
MHC1	`GraphChainer`	99.76% (+1.78%)	99.49% (+1.78%)
	`GraphAligner`	98.02%	97.74%
	`minigraph`	80.16%	78.81%
	`minichain`	30.30%	29.07%
Chr22	`GraphChainer`	99.74% (+1.51%)	99.31% (+1.34%)
	`GraphAligner`	98.26%	97.99%
	`minigraph`	86.01%	84.57%
	`minichain`	50.16%	49.13%
Chr1	`GraphChainer`	99.64% (+1.58%)	99.27% (+1.50%)
	`GraphAligner`	98.09%	97.79%
	`minigraph`	82.87%	81.25%
	`minichain`	38.73%	37.49%
AllChr	`GraphChainer`	99.61% (+1.89%)	99.24% (+1.83%)
	`GraphAligner`	97.76%	97.46%
	`minigraph`	77.71%	75.86%
	`minichain`	28.98%	27.85%
10H	`GraphChainer`	98.42% (+1.65%)	97.78% (+1.47%)
	`GraphAligner`	96.82%	96.37%
	`minigraph`	82.06%	81.08%
	`minichain`	84.32%	83.35%
95H	`GraphChainer`	94.06% (+1.50%)	92.54% (+1.22%)
	`GraphAligner`	92.67%	91.42%
	`minigraph`	63.27%	61.35%
	`minichain`	66.67%	64.79%

Table 13. Read length in correctly aligned reads with respect to the distance, for $\sigma_{truth} = 0.3$ (i.e., the edit distance between the truth sequence and the reported sequence can be up to 30% of the truth sequence length), and $\sigma_{read} = 0.3$ (i.e., the edit distance between the read and the reported sequence can be up to 30% of the read length) for simulated read sets. Percentages in parentheses are relative improvements w.r.t. `GraphAligner`.

Graph	Aligner	Good length	
		$\sigma_{truth} = 0.3$	$\sigma_{read} = 0.3$
LRC	`GraphChainer`	99.08% (+6.78%)	95.87% (+5.97%)
	`GraphAligner`	92.79%	90.47%
	`minigraph`	17.09%	14.61%
	`minichain`	1.38%	1.05%
MHC1	`GraphChainer`	99.00% (+5.97%)	96.76% (+5.10%)
	`GraphAligner`	93.42%	92.06%
	`minigraph`	21.88%	18.62%
	`minichain`	0.92%	0.70%
Chr22	`GraphChainer`	99.05% (+5.41%)	96.35% (+4.69%)
	`GraphAligner`	93.97%	92.03%
	`minigraph`	40.49%	37.04%
	`minichain`	29.55%	27.95%
Chr1	`GraphChainer`	98.39% (+4.99%)	95.58% (+4.03%)
	`GraphAligner`	93.72%	91.87%
	`minigraph`	27.20%	23.58%
	`minichain`	9.39%	8.72%
AllChr	`GraphChainer`	98.13% (+5.47%)	95.22% (+4.42%)
	`GraphAligner`	93.04%	91.17%
	`minigraph`	16.72%	13.87%
	`minichain`	1.65%	1.54%

Table 14. Read length in correctly aligned reads with respect to the distance, for $\sigma_{truth} = 0.3$ (i.e., the edit distance between the truth sequence and the reported sequence can be up to 30% of the truth sequence length), and $\sigma_{read} = 0.3$ (i.e., the edit distance between the read and the reported sequence can be up to 30% of the read length) for simulated read sets with error rate 5% as opposed to 15% shown in Table 13. Percentages in parentheses are relative improvements w.r.t. `GraphAligner`. 10H and 95H are the smallest and largest graphs used in the experiments of `minichain`, respectively.

Graph	Aligner	Good length	
		$\sigma_{truth} = 0.3$	$\sigma_{read} = 0.3$
LRC	`GraphChainer`	99.77% (+2.37%)	99.36% (+2.04%)
	`GraphAligner`	97.46%	97.37%
	`minigraph`	82.72%	81.67%
	`minichain`	27.44%	26.12%
MHC1	`GraphChainer`	99.88% (+2.46%)	99.45% (+2.40%)
	`GraphAligner`	97.48%	97.13%
	`minigraph`	84.71%	83.51%
	`minichain`	39.32%	38.00%
Chr22	`GraphChainer`	99.81% (+1.93%)	99.37% (+1.82%)
	`GraphAligner`	97.92%	97.59%
	`minigraph`	89.23%	88.02%
	`minichain`	56.52%	55.42%
Chr1	`GraphChainer`	99.69% (+2.07%)	99.27% (+2.04%)
	`GraphAligner`	97.68%	97.29%
	`minigraph`	86.69%	85.32%
	`minichain`	47.62%	46.22%
AllChr	`GraphChainer`	99.68% (+2.50%)	99.25% (+2.50%)
	`GraphAligner`	97.25%	96.83%
	`minigraph`	82.86%	81.22%
	`minichain`	38.18%	36.85%
10H	`GraphChainer`	97.92% (+2.03%)	97.19% (+1.89%)
	`GraphAligner`	95.97%	95.39%
	`minigraph`	78.85%	77.95%
	`minichain`	80.97%	80.05%
95H	`GraphChainer`	92.26% (+1.94%)	90.48% (+1.72%)
	`GraphAligner`	90.50%	88.96%
	`minigraph`	60.43%	58.30%
	`minichain`	63.37%	61.22%

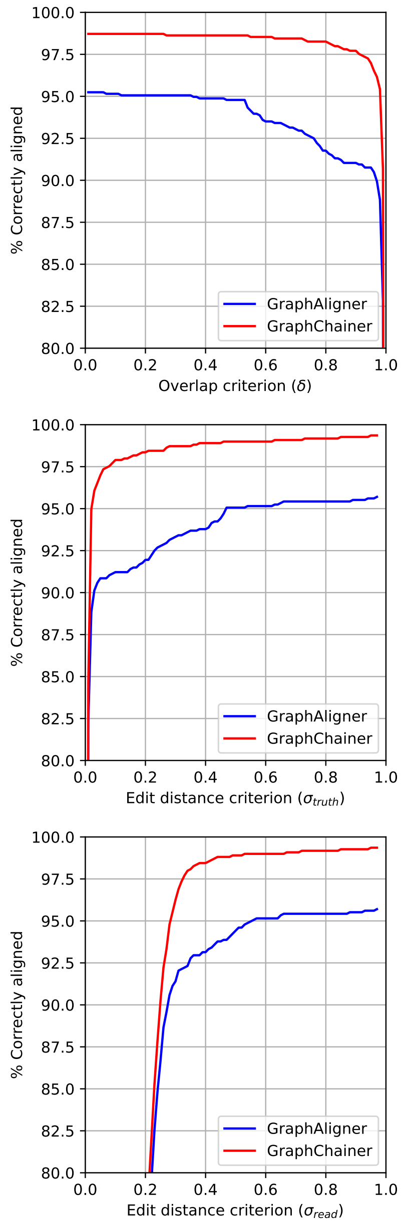

Fig. 5: Correctly aligned reads w.r.t overlap (top), truth sequence distance (middle) (middle) and read distance (bottom) for LRC on simulated reads (only `GraphAligner` and `GraphChainer`).

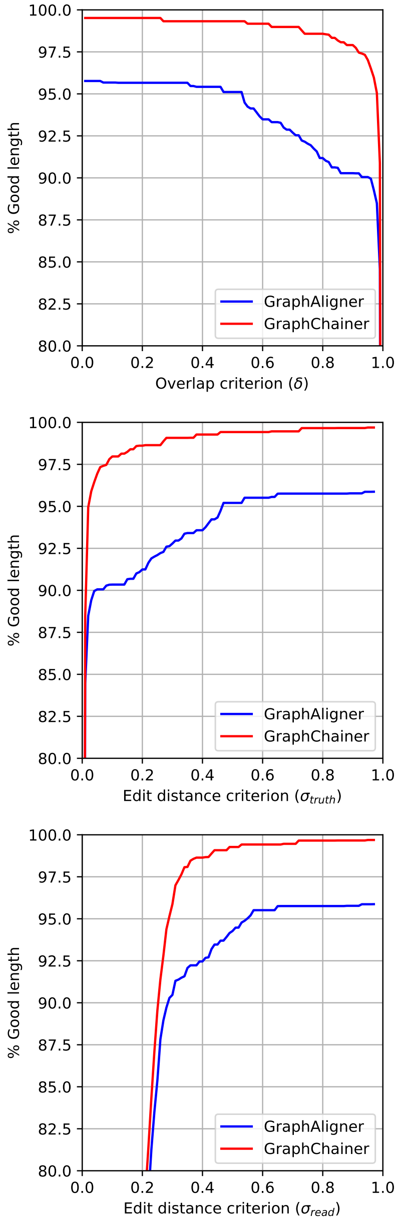

Fig. 6: Read length in correctly aligned reads w.r.t overlap (top), truth sequence distance (middle) and read distance (bottom) for LRC on simulated reads (only `GraphAligner` and `GraphChainer`).

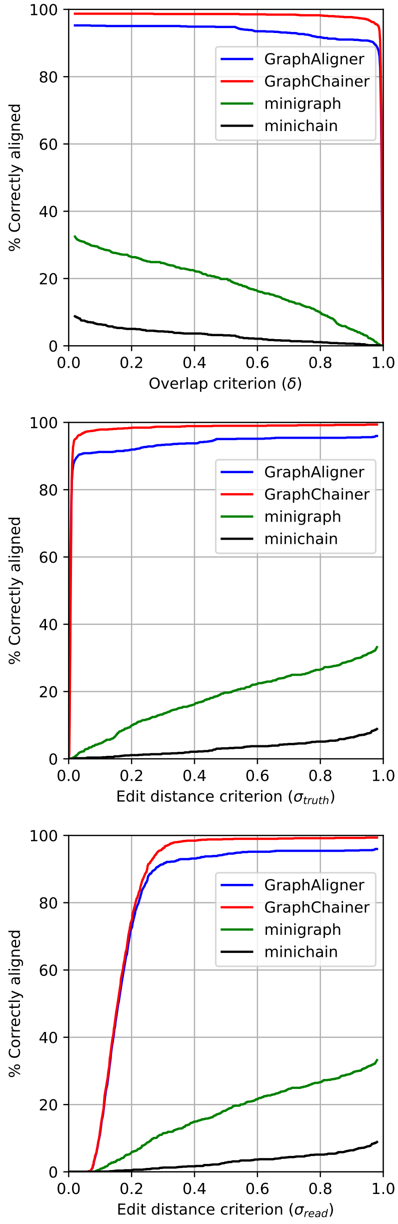

Fig. 7: Correctly aligned reads w.r.t overlap (top), truth sequence distance (middle) (middle) and read distance (bottom) for LRC on simulated reads.

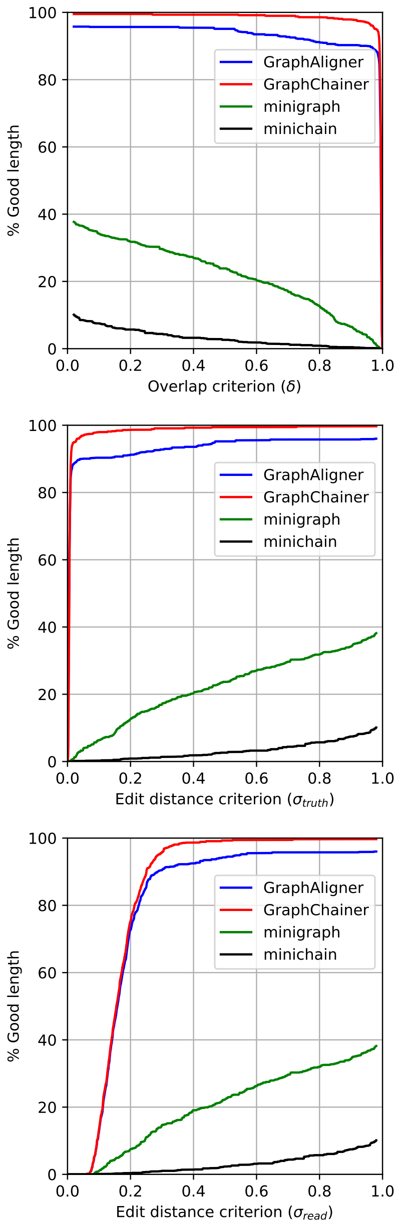

Fig. 8: Read length in correctly aligned reads w.r.t overlap (top), truth sequence distance (middle) and read distance (bottom) for LRC on simulated reads.

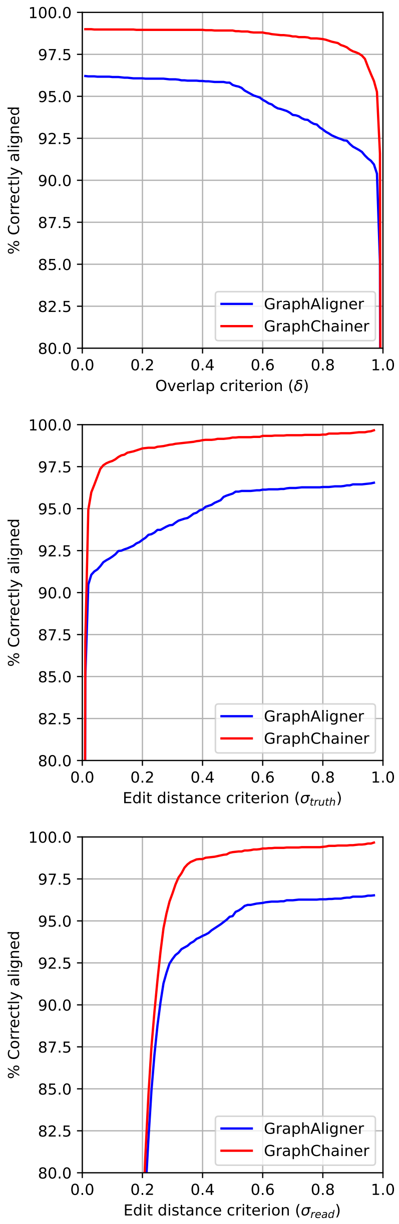

Fig. 9: Correctly aligned reads w.r.t overlap (top), truth sequence distance (middle) and read distance (bottom) for MHC1 on simulated reads (only `GraphAligner` and `GraphChainer`).

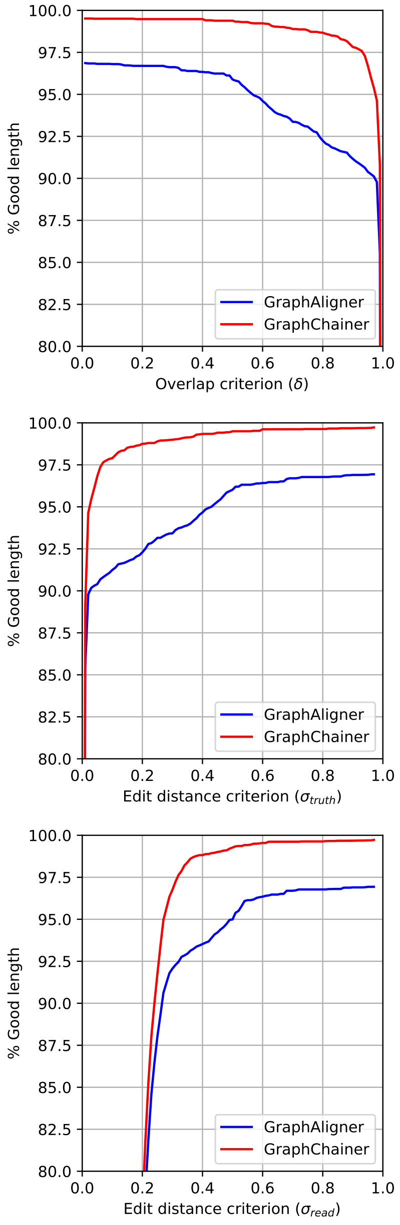

Fig. 10: Read length in correctly aligned reads w.r.t overlap (top), truth sequence distance (middle) and read distance (bottom) for MHC1 on simulated reads (only `GraphAligner` and `GraphChainer`).

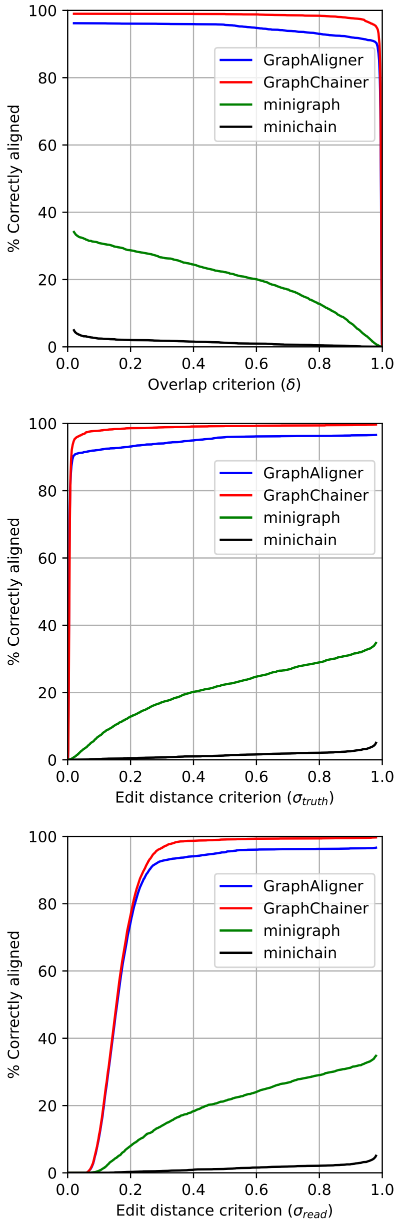

Fig. 11: Correctly aligned reads w.r.t overlap (top), truth sequence distance (middle) and read distance (bottom) for MHC1 on simulated reads.

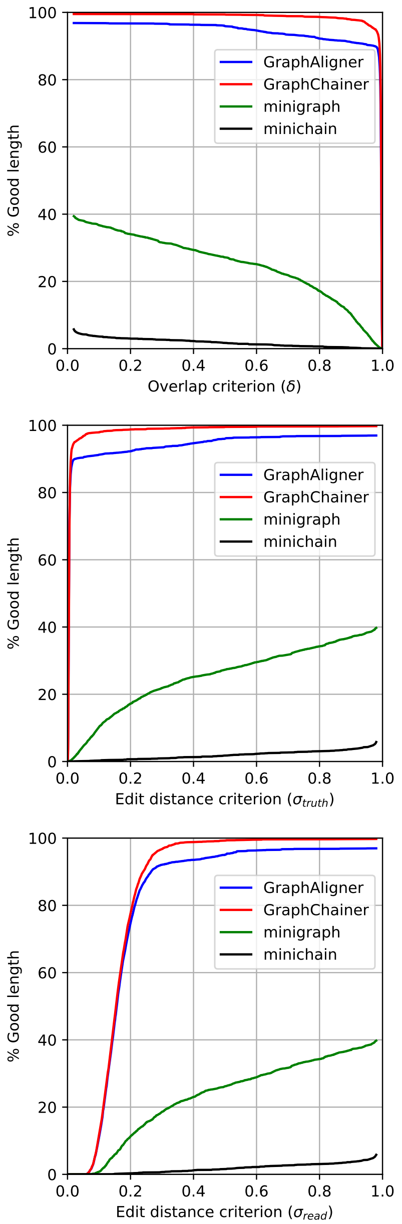

Fig. 12: Read length in correctly aligned reads w.r.t overlap (top), truth sequence distance (middle) and read distance (bottom) for MHC1 on simulated reads.

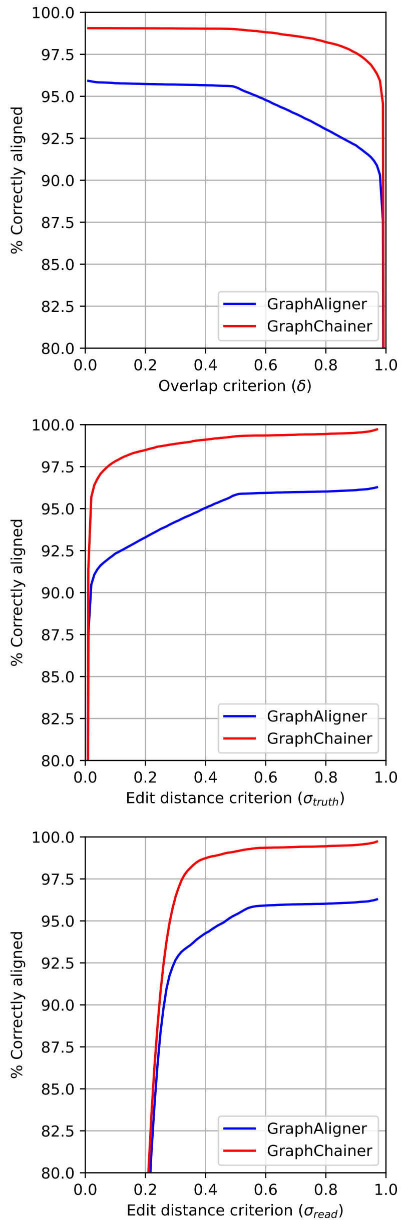

Fig. 13: Correctly aligned reads w.r.t overlap (top), truth sequence distance (middle) and read distance (bottom) for Chr22 on simulated reads (only `GraphAligner` and `GraphChainer`).

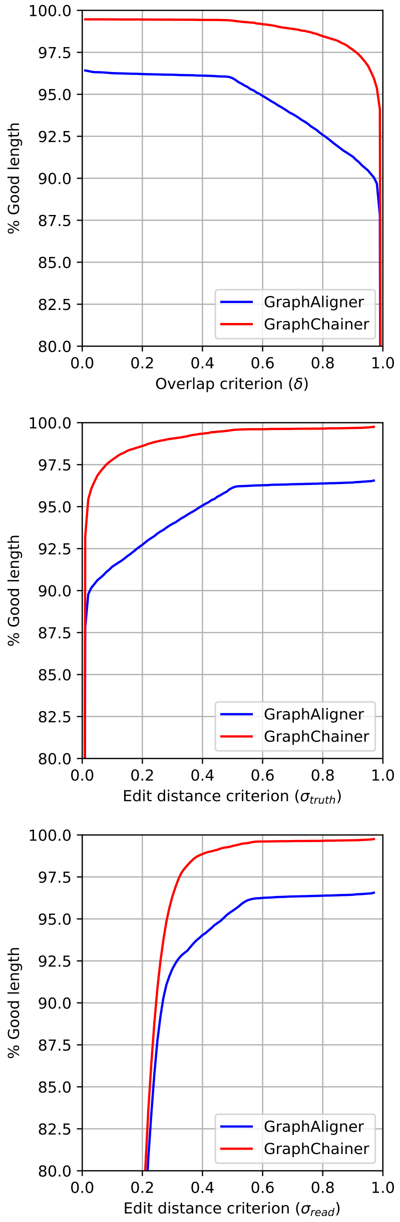

Fig. 14: Read length in correctly aligned reads w.r.t overlap (top), truth sequence distance (middle) and read distance (bottom) for Chr22 on simulated reads (only `GraphAligner` and `GraphChainer`).

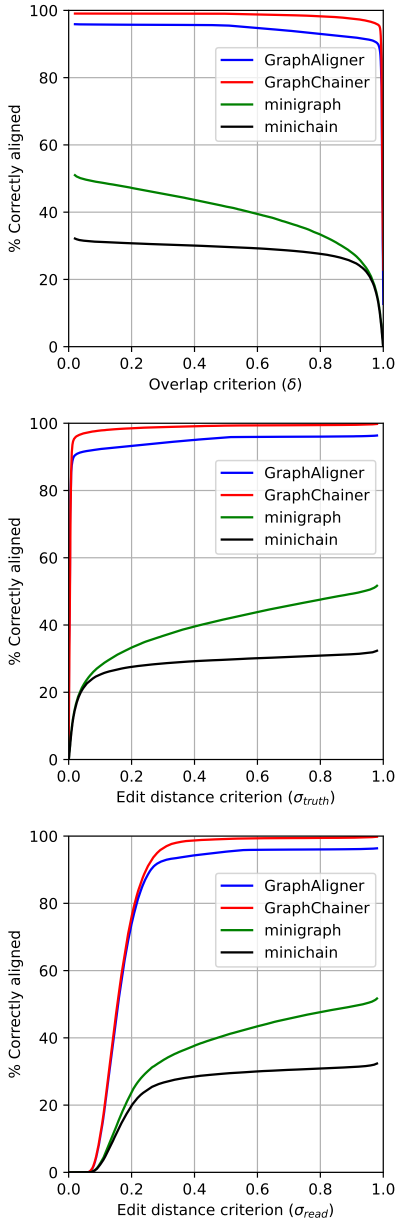

Fig. 15: Correctly aligned reads w.r.t overlap (top), truth sequence distance (middle) and read distance (bottom) for Chr22 on simulated reads.

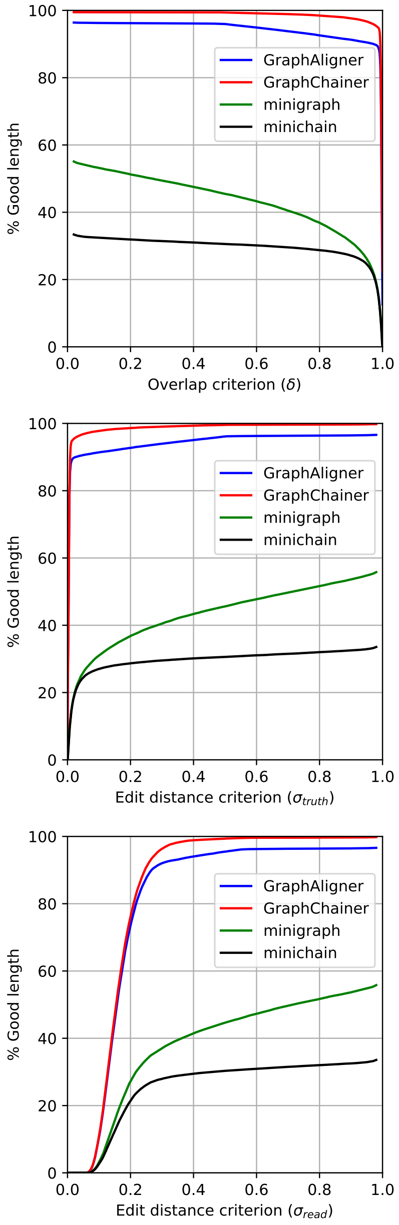

Fig. 16: Read length in correctly aligned reads w.r.t overlap (top), truth sequence distance (middle) and read distance (bottom) for Chr22 on simulated reads.

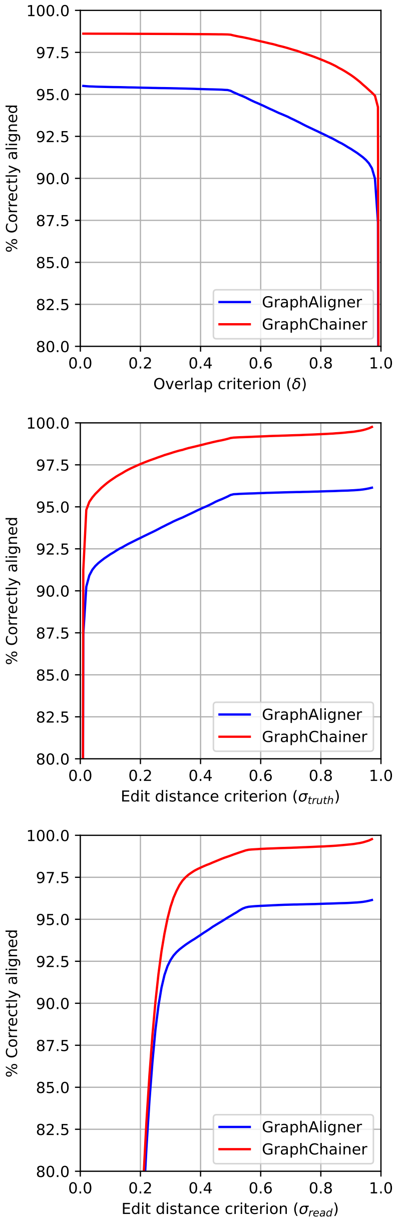

Fig. 17: Correctly aligned reads w.r.t overlap (top), truth sequence distance (middle) and read distance (bottom) for Chr1 on simulated reads (only `GraphAligner` and `GraphChainer`).

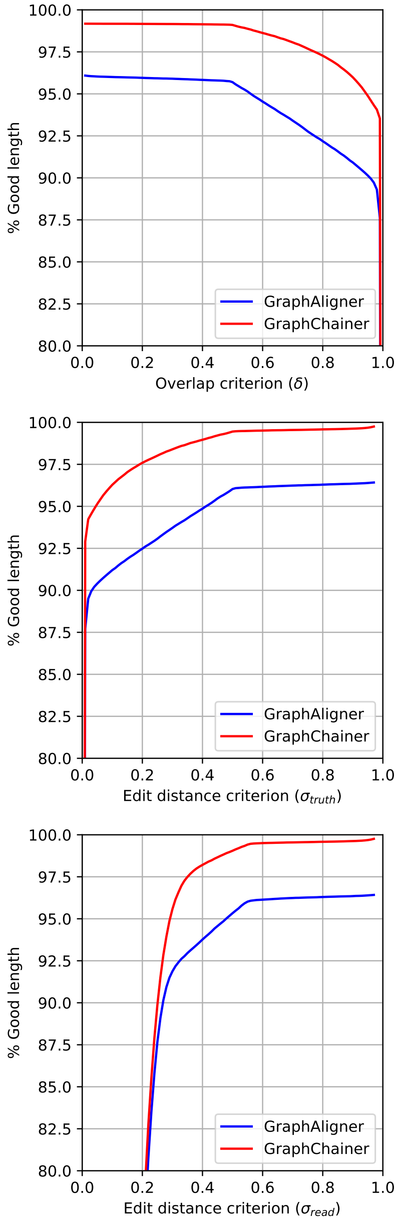

Fig. 18: Read length in correctly aligned reads w.r.t overlap (top), truth sequence distance (middle) and read distance (bottom) for Chr1 on simulated reads (only `GraphAligner` and `GraphChainer`).

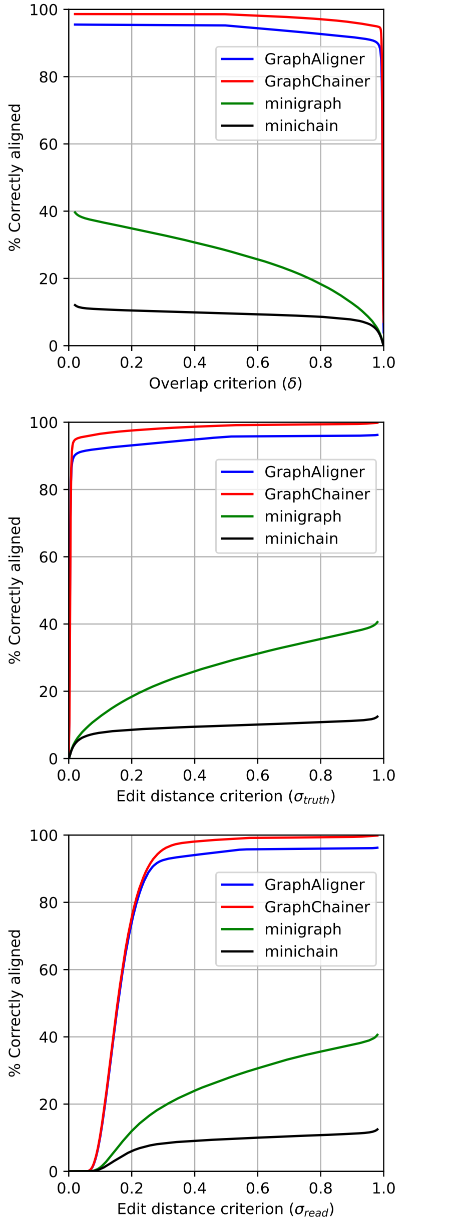

Fig. 19: Correctly aligned reads w.r.t overlap (top), truth sequence distance (middle) and read distance (bottom) for Chr1 on simulated reads.

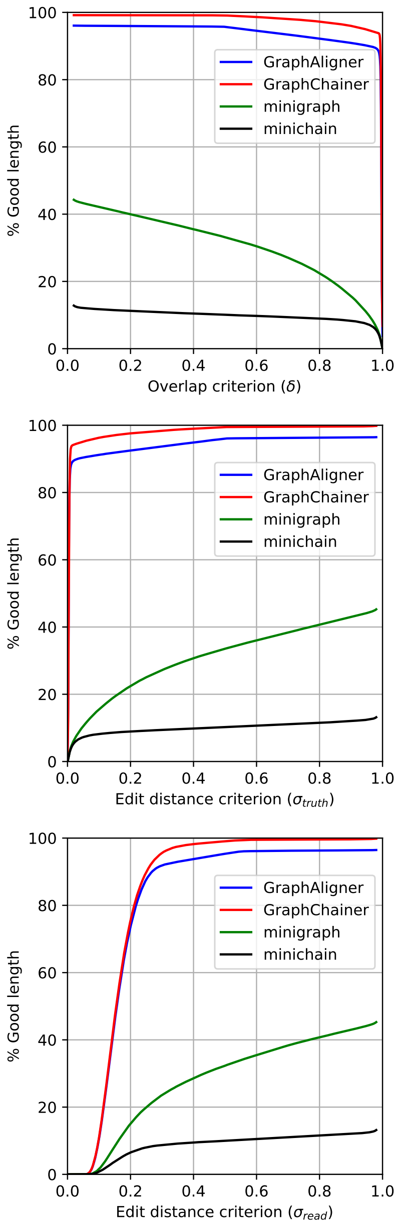

Fig. 20: Read length in correctly aligned reads w.r.t overlap (top), truth sequence distance (middle) and read distance (bottom) for Chr1 on simulated reads.

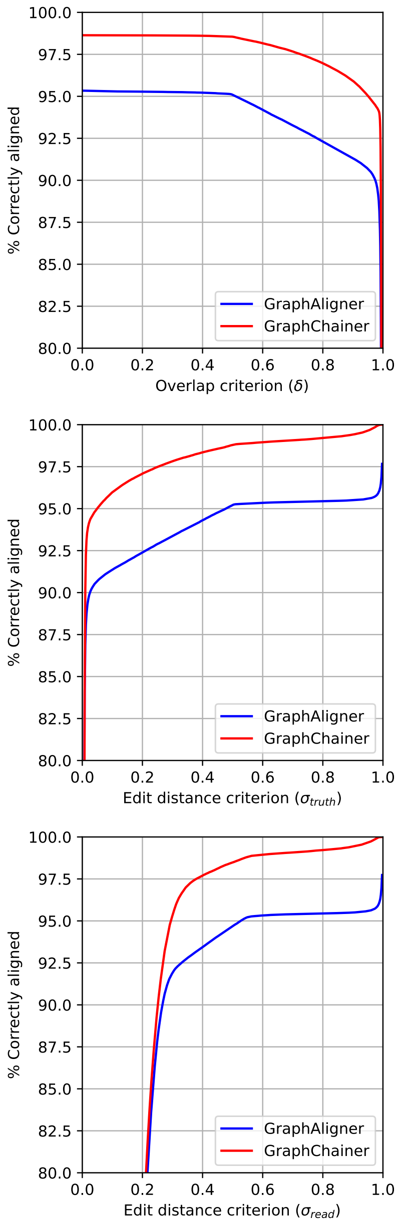

Fig. 21: Correctly aligned reads w.r.t overlap (top), truth sequence distance (middle) and read distance (bottom) for AllChr on simulated reads (only `GraphAligner` and `GraphChainer`).

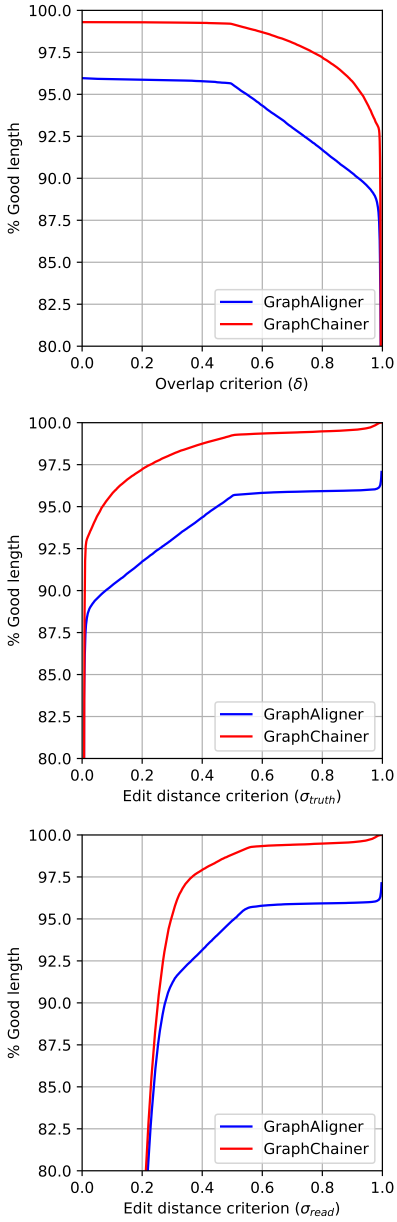

Fig. 22: Read length in correctly aligned reads w.r.t overlap (top), truth sequence distance (middle) and read distance (bottom) for AllChr on simulated reads (only `GraphAligner` and `GraphChainer`).

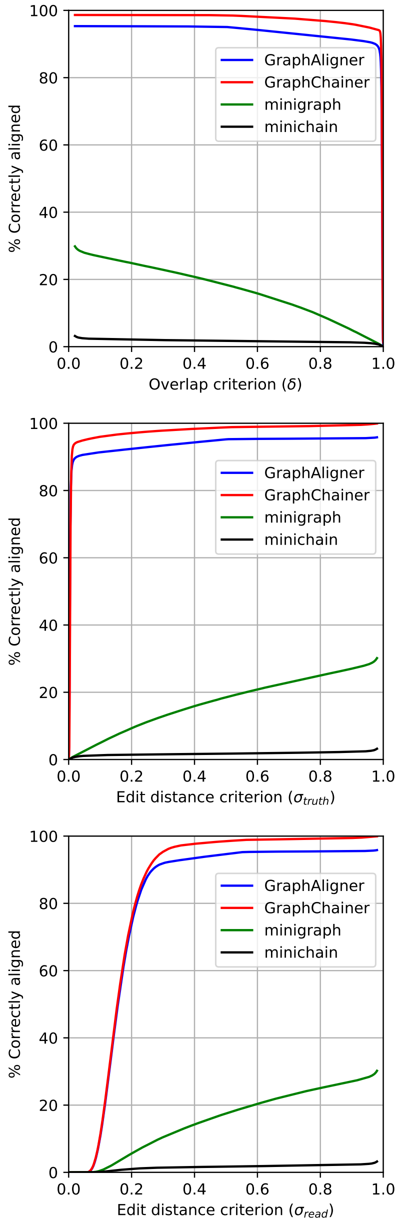

Fig. 23: Correctly aligned reads w.r.t overlap (top), truth sequence distance (middle) and read distance (bottom) for AllChr on simulated reads.

"output" — 2023/6/8 — 13:51 — page 27 — #27

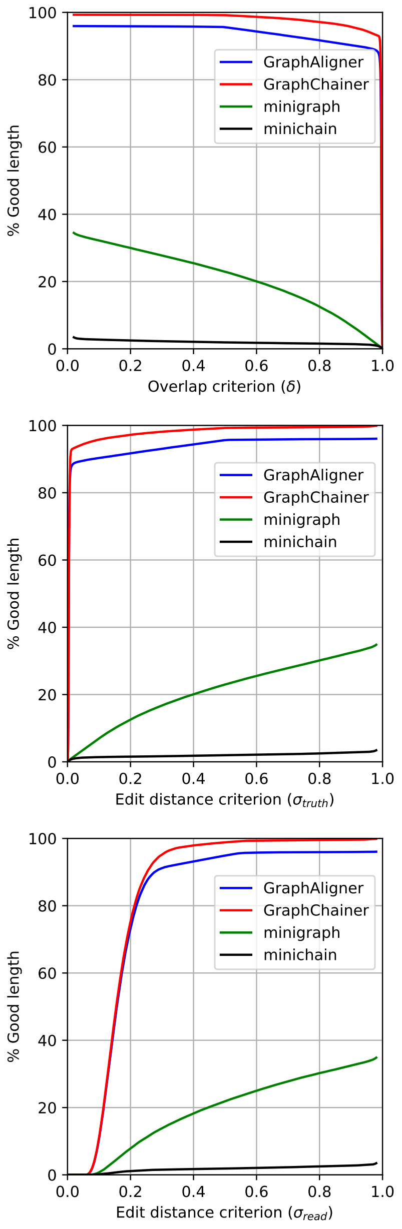

Fig. 24: Read length in correctly aligned reads w.r.t overlap (top), truth sequence distance (middle) and read distance (bottom) for AllChr on simulated reads.

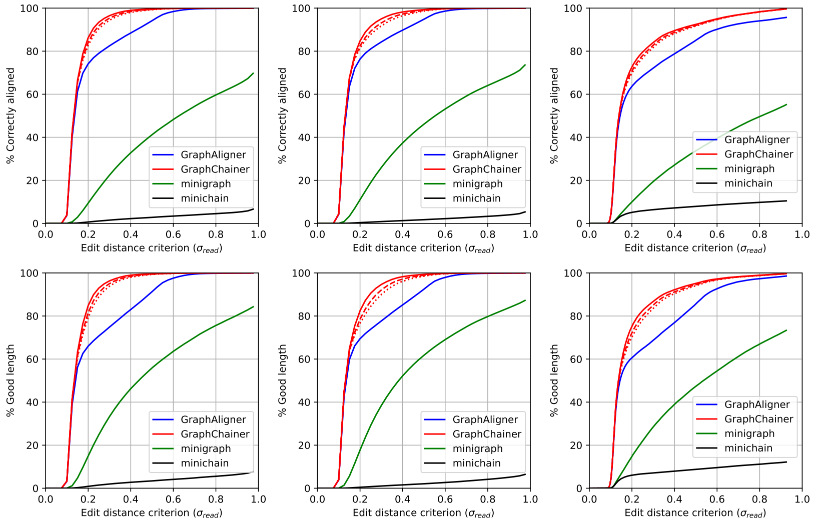

Fig. 25: Correctly aligned reads w.r.t. the read distance (top), and read length in correctly aligned reads (bottom), on Chr22 (left), Chr1 (center) and AllChr (right), for real PacBio CLR read sets.

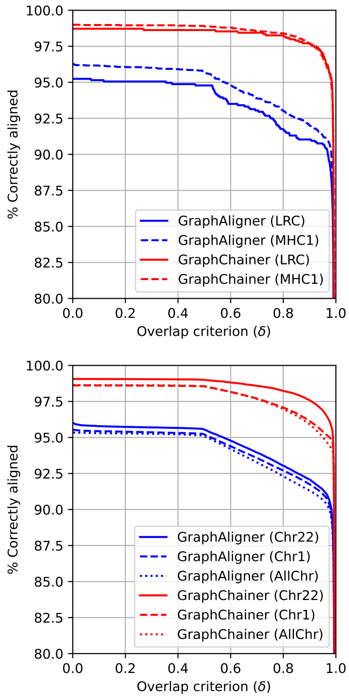

Fig. 26: Correctly aligned reads w.r.t overlap with ground truth on the simulated read sets (5% as opposed to 15% shown in Figure 2) for LRC (top solid), MHC1 (top dashed), Chr22 (bottom solid), Chr1 (bottom dashed) and AllChr (bottom dotted).

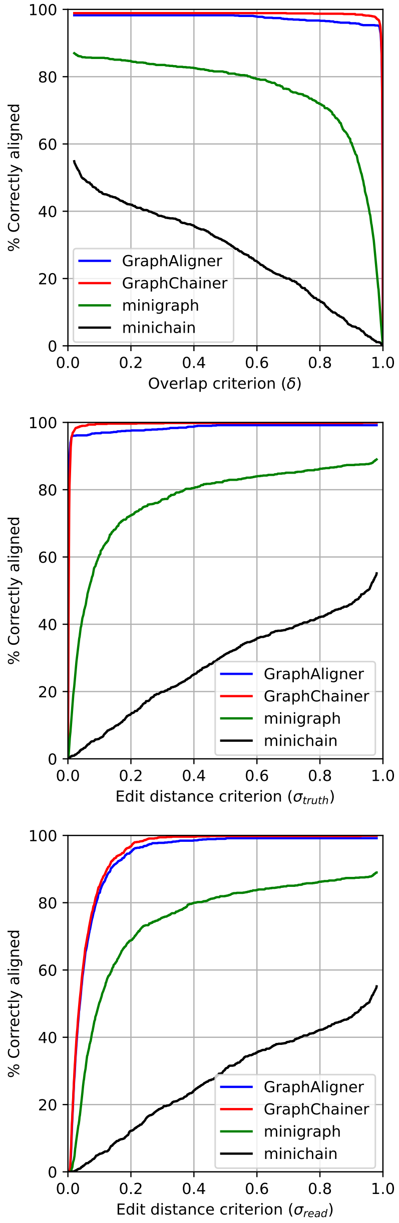

Fig. 27: Correctly aligned reads w.r.t overlap (top), truth sequence distance (middle) (middle) and read distance (bottom) for LRC on simulated reads with error rate 5% as opposed to 15% shown in Figure 7.

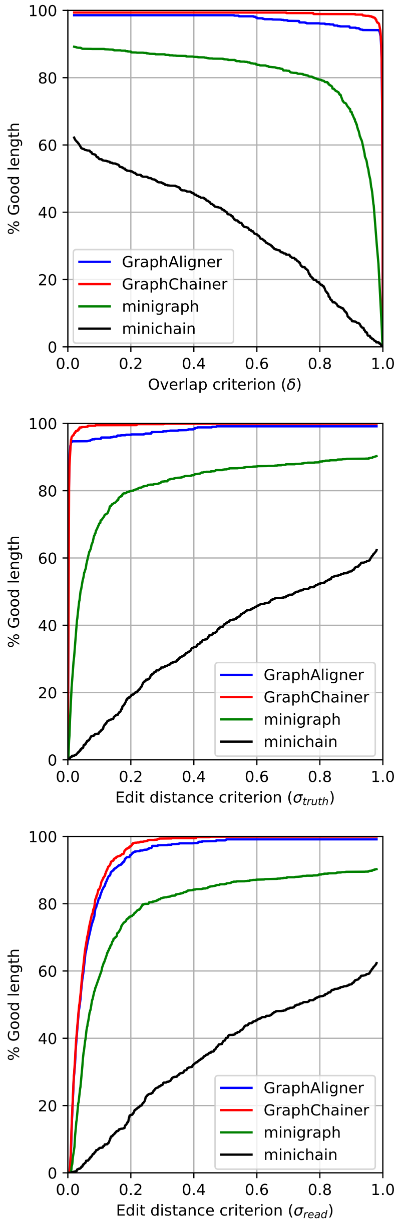

Fig. 28: Read length in correctly aligned reads w.r.t overlap (top), truth sequence distance (middle) and read distance (bottom) for LRC on simulated reads with error rate 5% as opposed to 15% shown in Figure 8.

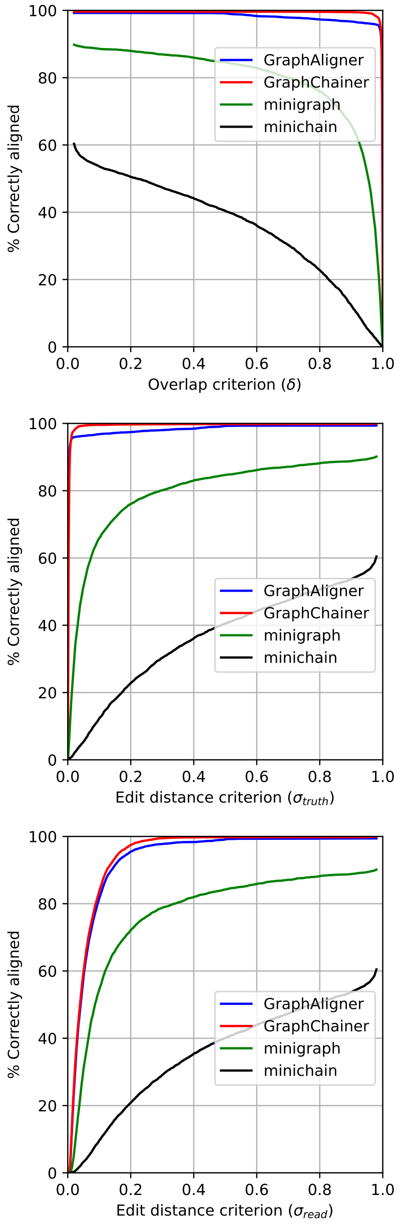

Fig. 29: Correctly aligned reads w.r.t overlap (top), truth sequence distance (middle) and read distance (bottom) for MHC1 on simulated reads with error rate 5% as opposed to 15% shown in Figure 11.

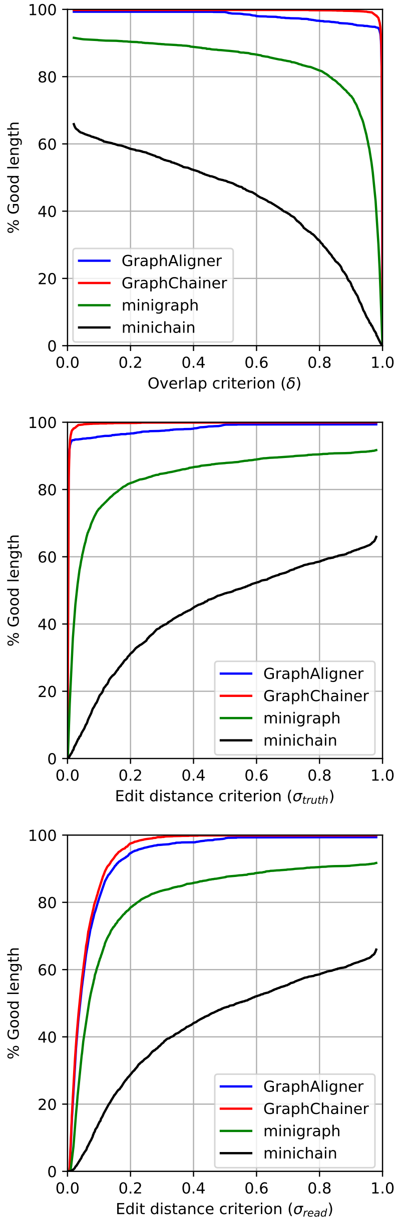

Fig. 30: Read length in correctly aligned reads w.r.t overlap (top), truth sequence distance (middle) and read distance (bottom) for MHC1 on simulated reads with error rate 5% as opposed to 15% shown in Figure 12.

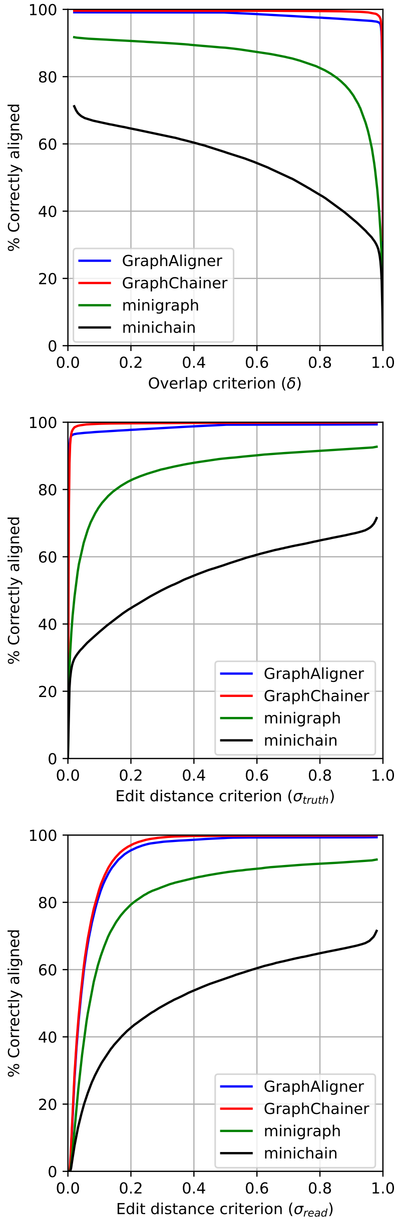

Fig. 31: Correctly aligned reads w.r.t overlap (top), truth sequence distance (middle) and read distance (bottom) for Chr22 on simulated reads with error rate 5% as opposed to 15% shown in Figure 15.

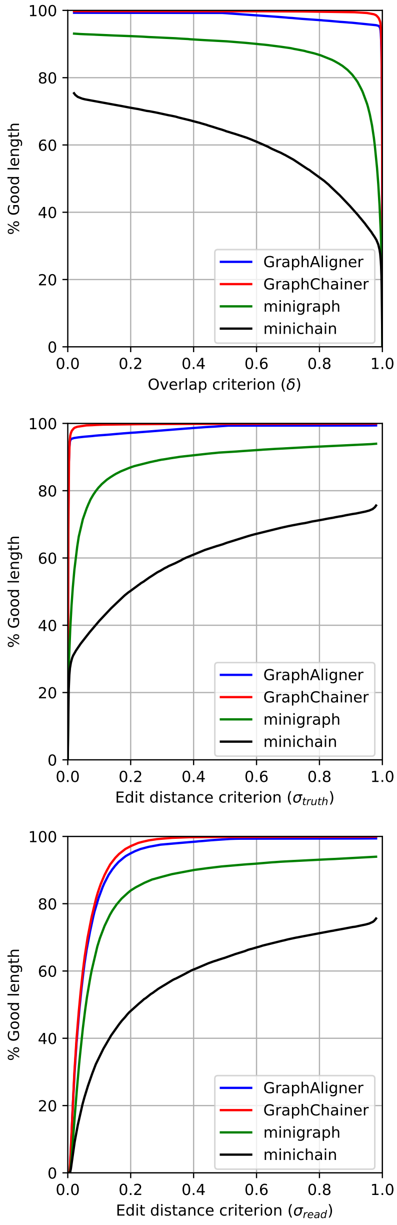

Fig. 32: Read length in correctly aligned reads w.r.t overlap (top), truth sequence distance (middle) and read distance (bottom) for Chr22 on simulated reads with error rate 5% as opposed to 15% shown in Figure 16.

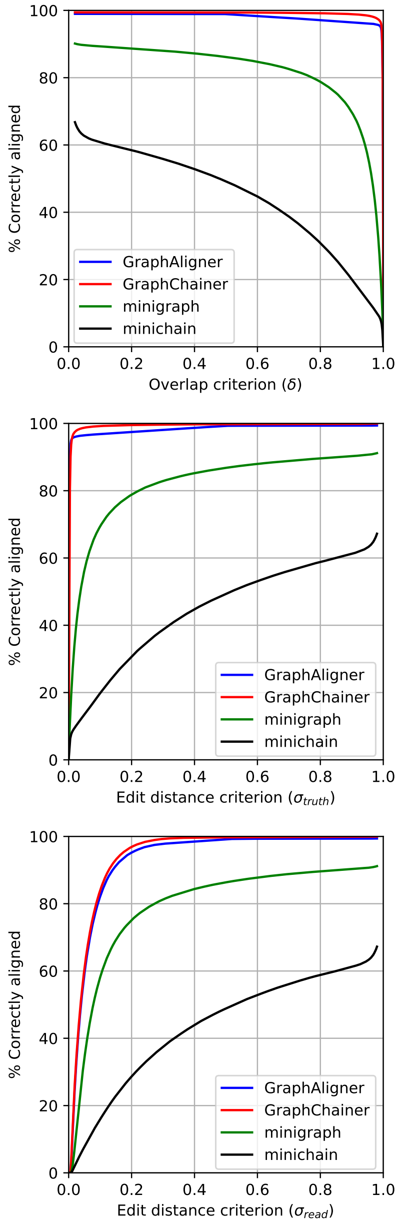

Fig. 33: Correctly aligned reads w.r.t overlap (top), truth sequence distance (middle) and read distance (bottom) for Chr1 on simulated reads with error rate 5% as opposed to 15% shown in Figure 19.

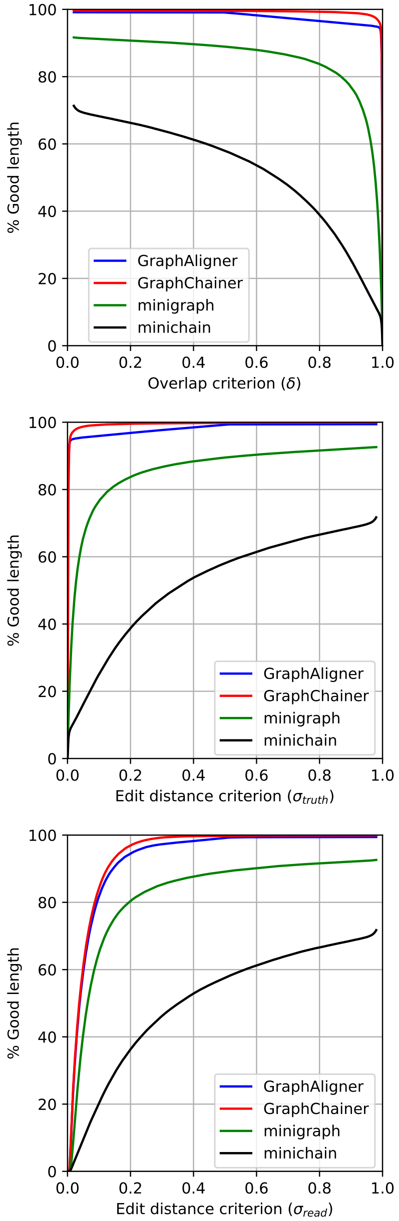

Fig. 34: Read length in correctly aligned reads w.r.t overlap (top), truth sequence distance (middle) and read distance (bottom) for Chr1 on simulated reads with error rate 5% as opposed to 15% shown in Figure 20.

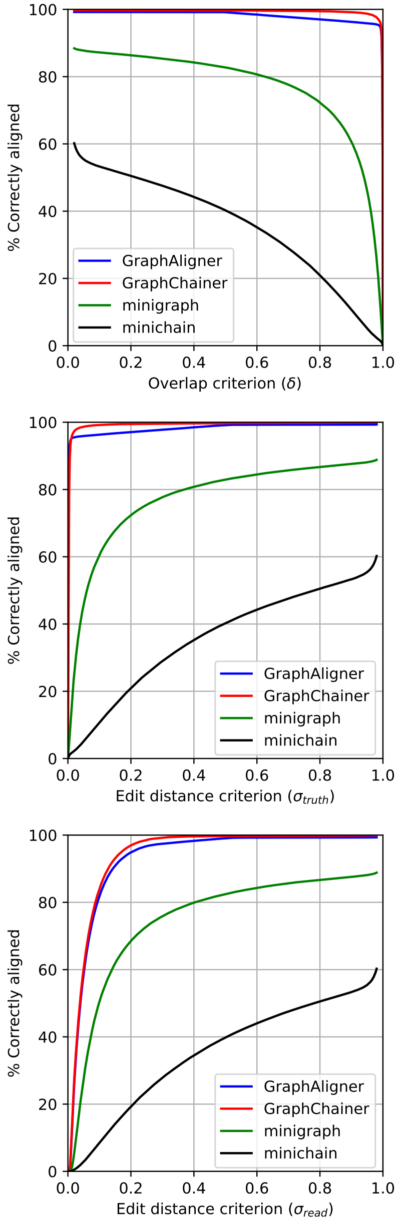

Fig. 35: Correctly aligned reads w.r.t overlap (top), truth sequence distance (middle) and read distance (bottom) for AllChr on simulated reads with error rate 5% as opposed to 15% shown in Figure 23.

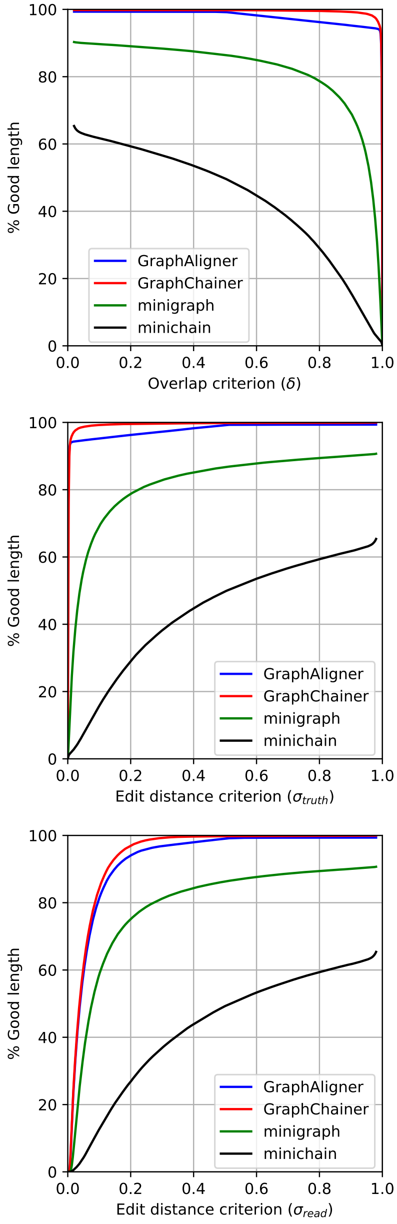

Fig. 36: Read length in correctly aligned reads w.r.t overlap (top), truth sequence distance (middle) and read distance (bottom) for AllChr on simulated reads with error rate 5% as opposed to 15% shown in Figure 24.

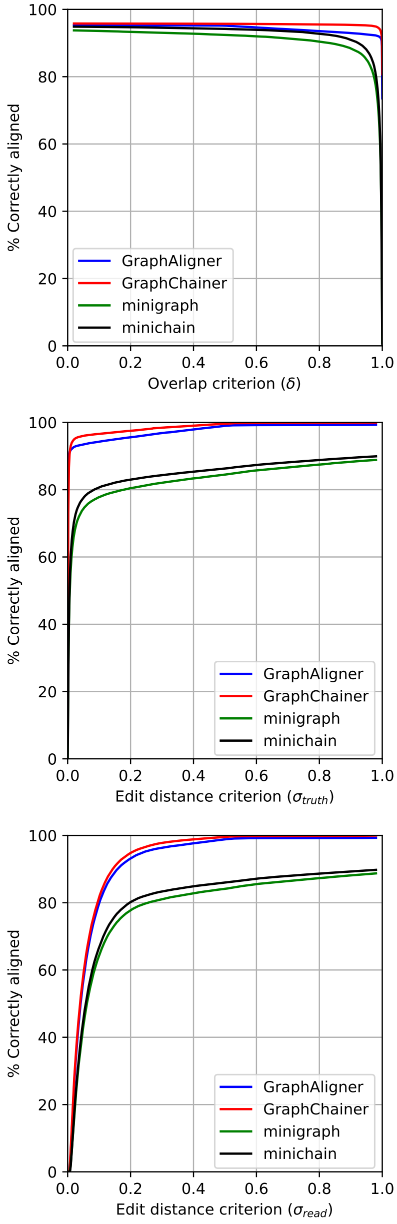

Fig. 37: Correctly aligned reads w.r.t overlap (top), truth sequence distance (middle) and read distance (bottom) for 10H on simulated reads with error rate 5%.

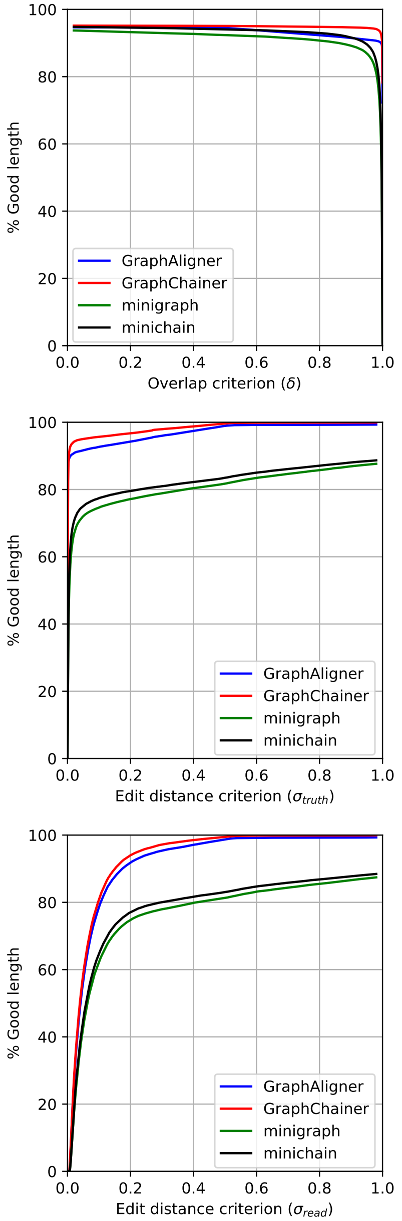

Fig. 38: Read length in correctly aligned reads w.r.t overlap (top), truth sequence distance (middle) and read distance (bottom) for 10H on simulated reads with error rate 5%.

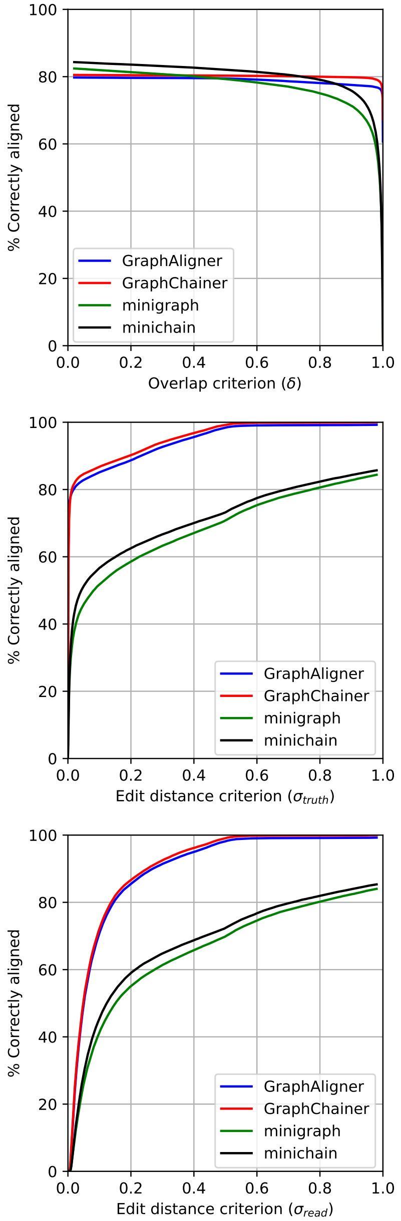

Fig. 39: Correctly aligned reads w.r.t overlap (top), truth sequence distance (middle) and read distance (bottom) for 95H on simulated reads with error rate 5%.

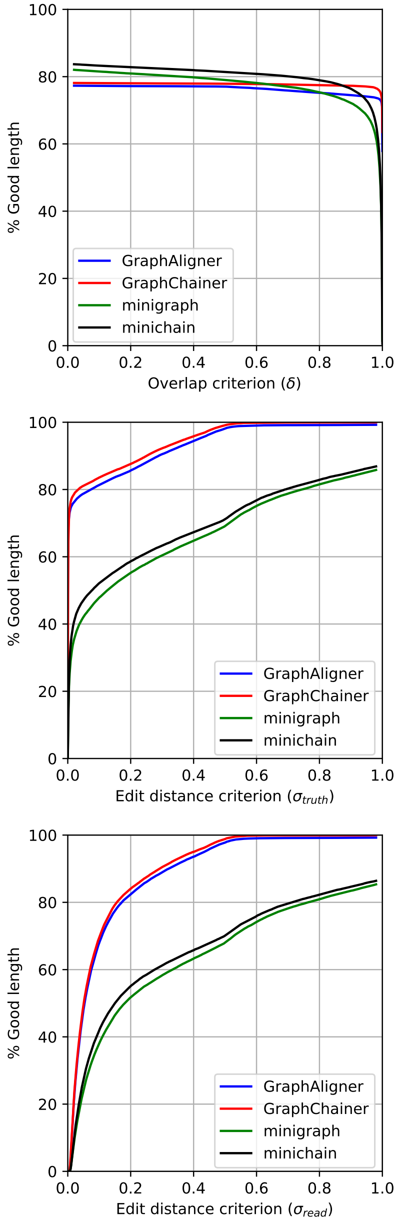

Fig. 40: Read length in correctly aligned reads w.r.t overlap (top), truth sequence distance (middle) and read distance (bottom) for 95H on simulated reads with error rate 5%.
